# eGFP-tagged Wnt-3a enables functional analysis of Wnt trafficking and signaling and kinetic assessment of Wnt binding to full-length Frizzled

**DOI:** 10.1074/jbc.RA120.012892

**Published:** 2020-05-07

**Authors:** Janine Wesslowski, Pawel Kozielewicz, Xianxian Wang, Haijun Cui, Hannes Schihada, Dominique Kranz, Pradhipa Karuna M, Pavel Levkin, Julia Christina Gross, Michael Boutros, Gunnar Schulte, Gary Davidson

**Affiliations:** 1 Institute of Biological and Chemical Systems–Functional Molecular Systems (IBCS-FMS), Karlsruhe Institute of Technology (KIT), Karlsruhe, Germany; 2 Section of Receptor Biology & Signaling, Department of Physiology & Pharmacology, Karolinska Institutet, Stockholm, Sweden; 3 Division of Signaling and Functional Genomics, German Cancer Research Center (DKFZ) and Heidelberg University, Heidelberg, Germany; 4 Hematology and Oncology/Developmental Biochemistry, University Medical Center Goettingen, Goettingen, Germany

**Keywords:** Wnt signaling, G protein–coupled receptor (GPCR), bioluminescence resonance energy transfer (BRET), fusion protein, membrane protein, trafficking, Frizzled, ligand binding, NanoBRET, NanoBiT

## Abstract

The Wingless/Int1 (Wnt) signaling system plays multiple, essential roles in embryonic development, tissue homeostasis, and human diseases. Although many of the underlying signaling mechanisms are becoming clearer, the binding mode, kinetics, and selectivity of 19 mammalian WNTs to their receptors of the class Frizzled (FZD_1–10_) remain obscure. Attempts to investigate Wnt-FZD interactions are hampered by the difficulties in working with Wnt proteins and their recalcitrance to epitope tagging. Here, we used a fluorescently tagged version of mouse Wnt-3a for studying Wnt-FZD interactions. We observed that the enhanced GFP (eGFP)-tagged Wnt-3a maintains properties akin to wild-type (WT) Wnt-3a in several biologically relevant contexts. The eGFP-tagged Wnt-3a was secreted in an evenness interrupted (EVI)/Wntless-dependent manner, activated Wnt/β-catenin signaling in 2D and 3D cell culture experiments, promoted axis duplication in *Xenopus* embryos, stimulated low-density lipoprotein receptor-related protein 6 (LRP6) phosphorylation in cells, and associated with exosomes. Further, we used conditioned medium containing eGFP-Wnt-3a to visualize its binding to FZD and to quantify Wnt-FZD interactions in real time in live cells, utilizing a recently established NanoBRET-based ligand binding assay. In summary, the development of a biologically active, fluorescent Wnt-3a reported here opens up the technical possibilities to unravel the intricate biology of Wnt signaling and Wnt-receptor selectivity.

In metazoans, cell-to-cell communication is predominantly controlled by the activation of specific cell surface receptors localized within the plasma membrane. Receptor-activating ligands are secreted from (paracrine) or presented on the surface (cell contact) of neighboring cells or presented as a humoral ligand with systemic effects (endocrine). Ligand-receptor interaction initiates conformational changes in the receptor to activate diverse intracellular signal transduction events that alter cellular behavior in a context-specific manner. The Wnt signaling system is an evolutionarily conserved, tightly regulated signaling network comprised of multiple transmembrane receptors and secreted ligands that play complex roles in embryonic development and tissue homeostasis (1–3). Ten different G protein–coupled receptors of the class F (FZD_1–10_) engage with 19 different lipidated Wnt proteins with unknown ligand-receptor selectivity to transduce Wnt signaling (1, 4–6). In addition to FZDs, coreceptors for Wnts are usually associated with specific branches of the pathway, such as low-density-lipoprotein-receptor-related proteins 5 and 6 (LRP5/6) for Wnt/β-catenin signaling (1). Pathway selectivity of Wnts is dictated by the relative availability of specific receptors, coreceptors, and Wnts, as well as the relative binding affinities of ligand-receptor interactions (7).

Various biochemical assays have been used to investigate the binding affinities of specific Wnt-FZD pairs, and *K_d_* values ranging from 5–100 nm have been reported in *Drosophila*, which have 5 Wnt and 4 FZD genes (7, 8). Such studies in higher vertebrates have proven more challenging because of the increased ligand-receptor diversity as well as difficulties associated with the biochemistry and lipophilicity of Wnt proteins resulting in low specific activity or a large proportion of nonspecific binding. Nevertheless, binding affinities ranging from 100 nm (Wnt-4/FZD_2_-CRD) down to 1.5 nm (Wnt-3a/FZD_8_-CRD) have also been reported (9, 10). All of these studies used membrane-tethered Wnts (7, 8) and/or the soluble, isolated cysteine-rich domain (CRD) of FZDs (8–10), which fail to fully recapitulate native conditions for Wnt interaction with the full-length FZD on live cells in real time.

Fluorescent labeling of proteins is a widely used and valued technique because of the range of biophysical and biochemical detection options it offers (11). However, the introduction of a large fluorescent moiety into a protein often compromises the stability and functionality of the target protein. A major limitation for the study of Wnt signaling and receptor binding originates from the lipophilicity of the Wnts themselves, which results in poor solubility and their recalcitrance to epitope tagging. Only a few attempts to generate functional, fluorescently tagged Wnt proteins have met with some degree of success, such as *Xenopus* Wnt-2b (12), *Xenopus* Wnt-5a (13), zebrafish Wnt-8 (14), and chick Wnt-1 (15), all of which are C-terminally tagged. Farin *et al.* elegantly demonstrated that successful epitope tagging of a mammalian Wnt is indeed possible after reporting that mice harboring an internal HA tag inserted at residue Q41 of the Wnt-3a gene were viable and could produce a fully functional, tractable Wnt-3a protein (16). Wnt-3a with Flag inserted at the same position (Wnt-3a–iFlag) is secreted from cells and binds to FZDs and LRP6 (17). More recently, an N-terminal GFP-tagged version of mouse Wnt-3a (GFP-Wnt-3a) was reported that is secreted from cells and shows partial activity (18). Despite these advancements, studies reporting quantitative analysis of the binding of a full-length, soluble Wnt protein to a full-length FZD protein on cells remain elusive.

Here, we describe a novel approach using full-length versions of Wnt-3a and FZDs to study the biology and biophysics of receptor engagement in real time using living cells. A combination of two recent developments enabled this: 1) the availability of a fluorescently tagged Wnt protein that is active, stable, and secreted into the medium of cultured cells, and 2) a highly sensitive proximity-based bioluminescence resonance energy transfer (BRET) assay, termed NanoBRET (19), which relies on resonance energy transfer from a cell surface-localized Nanoluciferase (Nluc) to a nearby enhanced GFP (eGFP). Compared with the standard luciferase from *Renilla reniformis*, Nluc from the deep-sea shrimp *Oplophorus gracilirostris* is smaller and brighter and has a spectrum reduced by ∼20 nm, all of which lends itself well to the precise study of ligand-receptor interactions in the complex milieu of living cells (19, 20). Our analysis of Wnt-3a binding to full-length FZD_4_ indicates that a high-affinity Wnt-FZD pair presents with a low-nanomolar equilibrium dissociation constant in living cells. Furthermore, we identify Afamin-dependent differences in the association of Wnt-3a with FZD_4_.

## Results

### Characterization of a functionally active eGFP-Wnt3a

**Figure 1.**
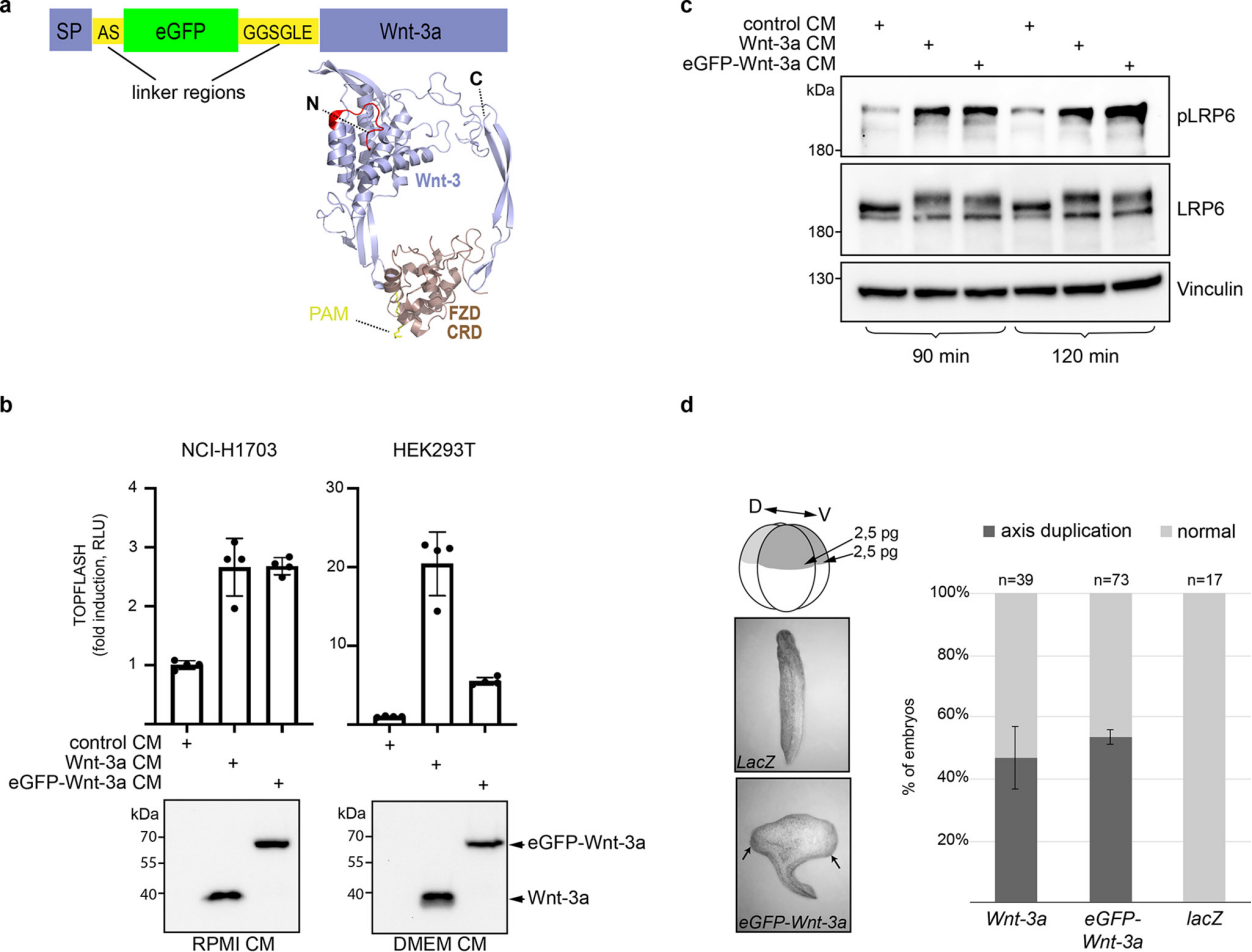
**Generation of functionally active eGFP-Wnt-3a.** *a*, Schematic overview of the eGFP-Wnt-3a fusion protein construct. For more details, refer to Fig. S1*a*. The protein complex model depicts human Wnt-8 (*violet*) and the mouse FZD_8_-CRD (*bronze*) based on the crystal structure (PDB entry 6AHY). The N terminus, where linker and eGFP are fused, is highlighted in *red*, and the C-terminal region is also indicated. The palmitoleoyl chain (PAM) is shown in *yellow*. The model was created in PyMOL (The PyMOL Molecular Graphics System, version 2.0; Schrödinger, LLC). *b*, Western blotting (*lower panels*, anti-Wnt-3a antibody) and TOPFLASH TCF reporter assays (*upper graphs*) showing presence of soluble Wnt-3a and eGFP-Wnt-3a in conditioned medium (CM) from stable L-cell lines and activity testing of the indicated CM in either NCI-H1703 or HEK293 cells, respectively. Note that CM is prepared using appropriate medium for the cell lines used (see Materials and methods for details). *Error bars* represent ± S.D. from means of 4 independent biological samples, represented as *solid dots*. Experiments were performed 3 times with similar results. *c*, Western blots showing Wnt-dependent phosphorylation and associated upshift of endogenous LRP6 protein bands from HEK293 cells after the addition of either Wnt-3a or eGFP-Wnt-3a L-cell CM, as indicated. The CM samples shown in *b* were used. Experiments were repeated at least three times with similar results. *d*, Axis duplication assay in *Xenopus laevis* embryos; 2.5 ng of either WT *Wnt-3a* or *eGFP-Wnt-3a* mRNA was injected equatorially in both ventral blastomeres of 4-cell stage embryos. Embryos (*n* defines total number of embryos evaluated) were scored for presence of a secondary axis the next day (stage 28), and *error bars* represent ± S.D. from means between three independent batches of injected embryos. Arrows indicate the two primary body axes.

Encouraged by recent reports of successful tagging of mouse Wnt-3a (16–18), we generated eGFP-Wnt-3a with the aim of utilizing this for ligand-receptor interaction studies. We fused eGFP directly to the N terminus of Wnt-3a, which projects away from the Wnt/FZD-CRD binding region (21, 22), using a short peptide linker (Fig. 1*a* and Fig. S1*a*). Our construct is similar to Flag-GFP-Wnt-3a generated by Takada *et al*. but lacks the Flag epitope tag (18). We generated a stably expressing L-cell line, which secretes biochemically stable eGFP-mWnt-3a into the medium similarly to WT Wnt-3a (Fig. 1*b*, *lower panels*, and Fig. S1*b*). When the conditioned medium (CM) is tested for functional activity using T-cell factor (TCF) reporter assays, eGFP-Wnt-3a behaves like WT Wnt-3a when using NCI-H1703 cells (Fig. 1*b*, *upper graph*). H1703 cells were chosen because they display strong Wnt/β-catenin signaling activity (23) and distinct cell surface expression of overexpressed Wnt receptors. Using HEK293 cells, however, eGFP-Wnt-3a shows only ≈20% activity of WT Wnt-3a (Fig. 1*b*, *upper right graph*). Transfection of LRP6 increased the basal Wnt activity of HEK293 cells and, under these conditions, the activity of eGFP-Wnt-3a relative to that of Wnt-3a increased from ≈20% to ≈60% (Fig. S1*d*). We next tested the ability of eGFP-Wnt-3a CM to induce LRP6 phosphorylation, which occurs in response to acute pathway stimulation (24, 25). Similar to Wnt-3a CM, the addition of eGFP-Wnt-3a CM to either HEK293 cells (Fig. 1*c*) or NCI-H1703 cells (Fig. S1, *d–e*) promotes phosphorylation of endogenous LRP6, which is accompanied by the characteristic upshift of the mature, cell surface LRP6 protein band (Fig. 1*c* and Fig. S1*d*). Further, we validated the activity of eGFP-Wnt-3a *in vivo* in *Xenopus* embryos, which provides a physiologically relevant model system for assessing Wnt activity (26). Duplication of the primary embryonic axis is well known to be robustly induced by Wnts (27), and mouse eGFP-Wnt-3a displays activities similar to those of untagged Wnt-3a in the axis duplication assay (Fig. 1*d*). Wnt-3a/eGFP-Wnt-3a CM was also prepared using transiently transfected HEK293F suspension cells in serum-free medium (Fig. S1*e*). The serum component Afamin binds to Wnt-3a and aids its secretion and/or release from cells into serum-free medium (28), and Afamin also improved eGFP-Wnt-3a secretion (Fig. S1*f*). Taken together, these results confirm that synthesis, secretion, stability, and signaling activity of eGFP-Wnt-3a are largely preserved upon fusion of the 25-kDa eGFP protein to the N terminus of Wnt-3a.

### eGFP-Wnt3a is secreted in an EVI-dependent manner and associates with exosomes

**Figure 2.**
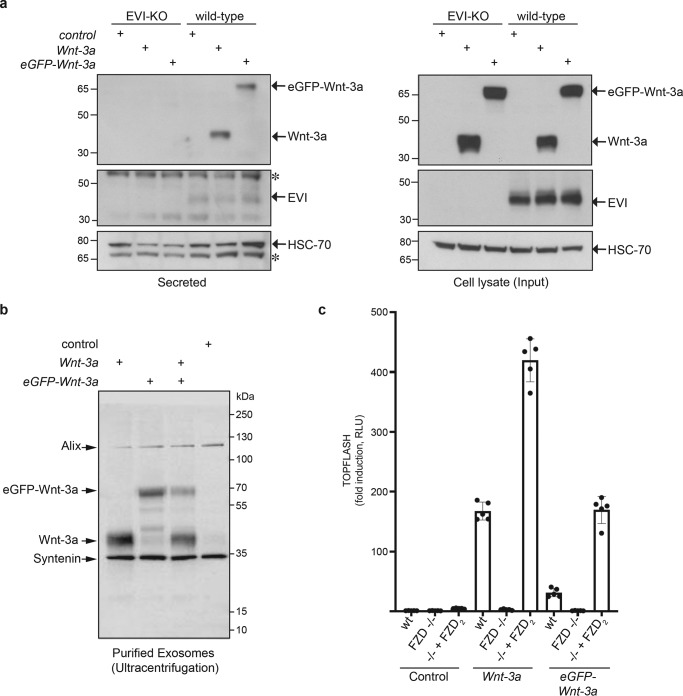
**EVI-dependent eGFP-Wnt-3a secretion and association of eGFP-Wnt-3a with exosomes.** *a*, Western blots of indicated proteins from medium (secreted) and corresponding cell lysates from WT HEK293T cells or EVI knockout HEK293T cells (HEK293T EVI-KO2.9), transfected as indicated. *Asterisks* indicate nonspecific bands. *b*, Western blot analysis of proteins from purified exosomes derived from HEK293 cells transfected as indicated with *Wnt-3a*, *eGFP-Wnt-3a*, or a 1:1 combination of both. Exosomes were purified by ultracentrifugation (see Materials and methods for details). The exosome marker proteins Alix and Syntenin are indicated. *c*, TCF reporter assay using either WT HEK293T cells or ΔFZD_1,2,4,5,7,8_ HEK293T cells transfected with FZD_2_, FLUC, RLUC in combination with the control (pcDNA), Wnt-3a, or eGFP-Wnt-3a. Error bars represent means ± S.D. from 5 independent biological samples, represented as *solid dots*. The experiment was performed 2 times with similar results.

Wnt proteins are acylated in the endoplasmic reticulum by the palmitoyltransferase porcupine (29), and the Wnt-specific chaperone EVI/Wntless mediates subsequent transport through the secretory pathway, which is necessary for correct cell surface transportation and release of lipidated Wnts from cells (30, 31). Since eGFP-Wnt-3a is efficiently secreted from mammalian cells, we investigated whether secretion depends on EVI/Wntless using ΔEVI mutant cells. Expression levels of WT and eGFP-Wnt-3a are similar; however, neither is secreted in ΔEVI mutant HEK293 cells (Fig. 2*a*). Another characteristic of lipidated Wnt proteins is their trafficking in exosomes, which is one mechanism that accounts for Wnt secretion (32, 33). In a further confirmation of the functional similarity between WT and fluorescently tagged Wnt-3a, exosomes purified from conditioned medium associate with both Wnt-3a and eGFP-Wnt-3a (Fig. 2*b* and Fig. S2). This was the case for exosomes purified using either ultracentrifugation (Fig. 2*b* and Fig. S2) or magnetic-activated cell sorting antibody-based sorting (Fig. S2). Exosomes were prepared from either HEK293 adherent cells in serum-containing medium (Fig. 2*b*) or HEK293F suspension cells in serum-free medium (Fig. S2).

### *eGFP-Wnt-3a acts through FZDs to mediate WNT/*β*-catenin signaling*

Wnt-3a acts through FZDs and LRP5/6 to transduce Wnt/β-catenin signaling manifested in the phosphorylation of LRP5/6, phosphorylation of the scaffold protein Disheveled (DVL), stabilization of β-catenin, and transcriptional regulation of TCF/LEF-dependent genes. Although endogenously expressed FZDs in HEK293 cells are sufficient to mediate WNT/β-catenin pathway activation, removal of FZD expression blunts Wnt-induced signaling (34–37). To define the role of FZDs in the eGFP-Wnt-3a-induced WNT/β-catenin response, we compared the transcriptional response induced by untagged Wnt-3a and eGFP-Wnt-3a in WT HEK293 cells or HEK293 cells lacking FZD_1,2,4,5,7,8_ (35). The complete absence of a Wnt response in FZD mutant HEK293 cells emphasizes that Wnt/β-catenin signaling activated by eGFP-Wnt-3a is transduced in an FZD-dependent manner (Fig. 2*c*). Transfection of FZD_2_ in the FZD mutant cells restores the ability of both untagged Wnt-3a and eGFP-Wnt-3a to transduce WNT/β-catenin signaling (Fig. 2*c*).

### *mCherry-Wnt-3a activates Wnt/*β*-catenin signaling at a distance from its source*

**Figure 3.**
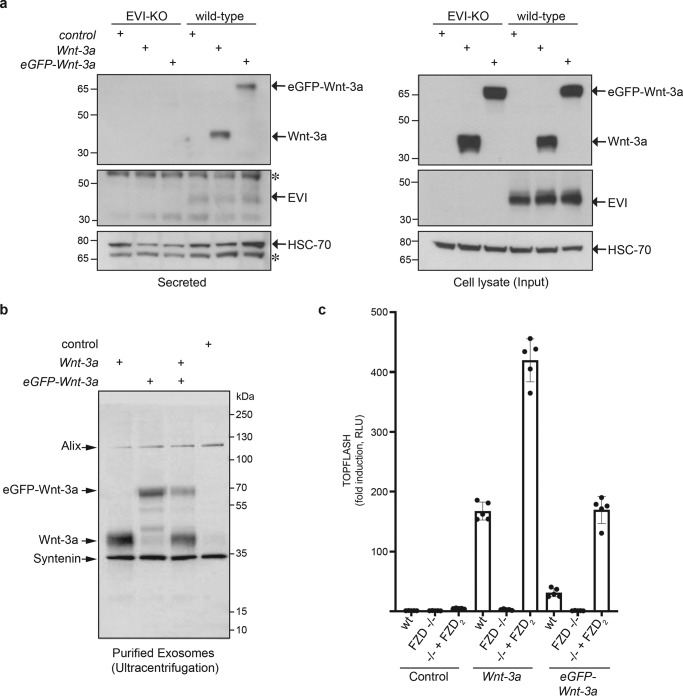
**Diffusion of mCherry-Wnt-3a within spheroids.** *a*, TOPFLASH TCF reporter assay showing the comparative activities of mCherry-Wnt-3a and eGFP-Wnt-3a when applied to HEK293 cells as L-cell conditioned medium (CM). *Error bars* represent ± S.D. from mean of 4 independent biological samples, represented as *solid dots*. The experiment was performed 2 times with similar results. Western blot analysis of eGFP-Wnt-3a and mCherry-Wnt-3a proteins in the CM is shown in the *lower panel*. *b*, HEK293T cell spheroid fusion assay for visualization of paracrine Wnt/β-catenin signaling. *mCherry-Wnt-3a-* or control *lacZ*-transfected HEK293 cells were used to form spheroids, which were then combined with spheroids prepared from a stable TOP-GFP HEK293 reporter cell line. Twenty-four or 48 h later, merged spheroids were imaged using laser scanning confocal microscopy for activation of Wnt/β-catenin signaling in the TOP-GFP reporter cells (*green* cells). The two *lower arrows* mark mCherry-Wnt3a-producing cells flanking a highly activated TOP-GFP reporter cell (*smaller upper arrow*). BF, bright-field image showing fused spheroids with the *dashed line* demarking the border between the Wnt secreting/control cells and the TOP-GFP reporter cells (TOP). The presented images are representative of findings obtained from 3 independent experiments. *c*, Bar graph showing the relative fluorescent intensities measured in the TOP‐GFP spheroids shown in *b*, calculated from at least 9 merged spheroids under each of the assays conditions. Error bars represent means ± S.D. from 10 independent fluorescence intensity values, represented as solid dots. The fluorescence intensity of the 24-h control TOP-GFP spheroids is set to 1.

Extracellular dispersal of Wnt proteins contributes to signaling events (38), and it is reported that Wnt-3a forms homotrimeric complexes, with dynamic formation of larger structures controlling the range with which Wnt proteins can diffuse through the extracellular space (18). To study the diffusion of our fluorescently tagged Wnt-3a, we developed a controllable, microdroplet-based cell spheroid formation and fusion assay (see Materials and methods for details), which provides a visual readout of Wnt paracrine signaling. For the fluorescently tagged Wnt-3a, spheroids are prepared from HEK293 cells transfected with mCherry-Wnt-3a, which has an activity similar to that of eGFP-Wnt-3a (Fig. 3*a*). For the Wnt/β-catenin signaling readout, we prepared spheroids of TOP-GFP HEK293 reporter cells, representing a TCF activity-reporting fluorescent reporter assay (see Supporting information for details). Confocal imaging of spheroids was performed 24 h or 48 h after their fusion, and the activation of TCF activity evoked by mCherry-Wnt3a-secreting cells is evident (Fig. 3*b*). The strongest TOP-GFP reporter activation is detected in cells that are in direct contact with the mCherry-Wnt-3a-expressing cells (see *arrows* in Fig. 3*b*, 24 h); however, distinct activation of TOP-GFP is observed in all spheroid cells, including those located several cell diameters away from mCherry-Wnt-3a-expressing cells. Background activation of fluorescence in TOP-GFP spheroids is low at the 24-h time point, and fluorescence levels are also similar after their fusion with control spheroids prepared from LacZ-transfected cells (Fig. 3*b*, 24 h), which confirms specific and mCherry-Wnt-3a-dependent activation. At the 48-h time point, background TOP-GFP reporter fluorescence increased only marginally in the control spheroid fusions, whereas the fluorescence intensity in TOP-GFP spheroids fused to mCherry-Wnt-3a-producing spheroids increased substantially (Fig. 3, *b* and *c*). These results indicate that fluorescently tagged Wnt-3a can readily diffuse between the tightly packed cells in the spheroids to signal at a distance.

### eGFP-Wnt3a interaction with FZD-mCherry

**Figure 4.**
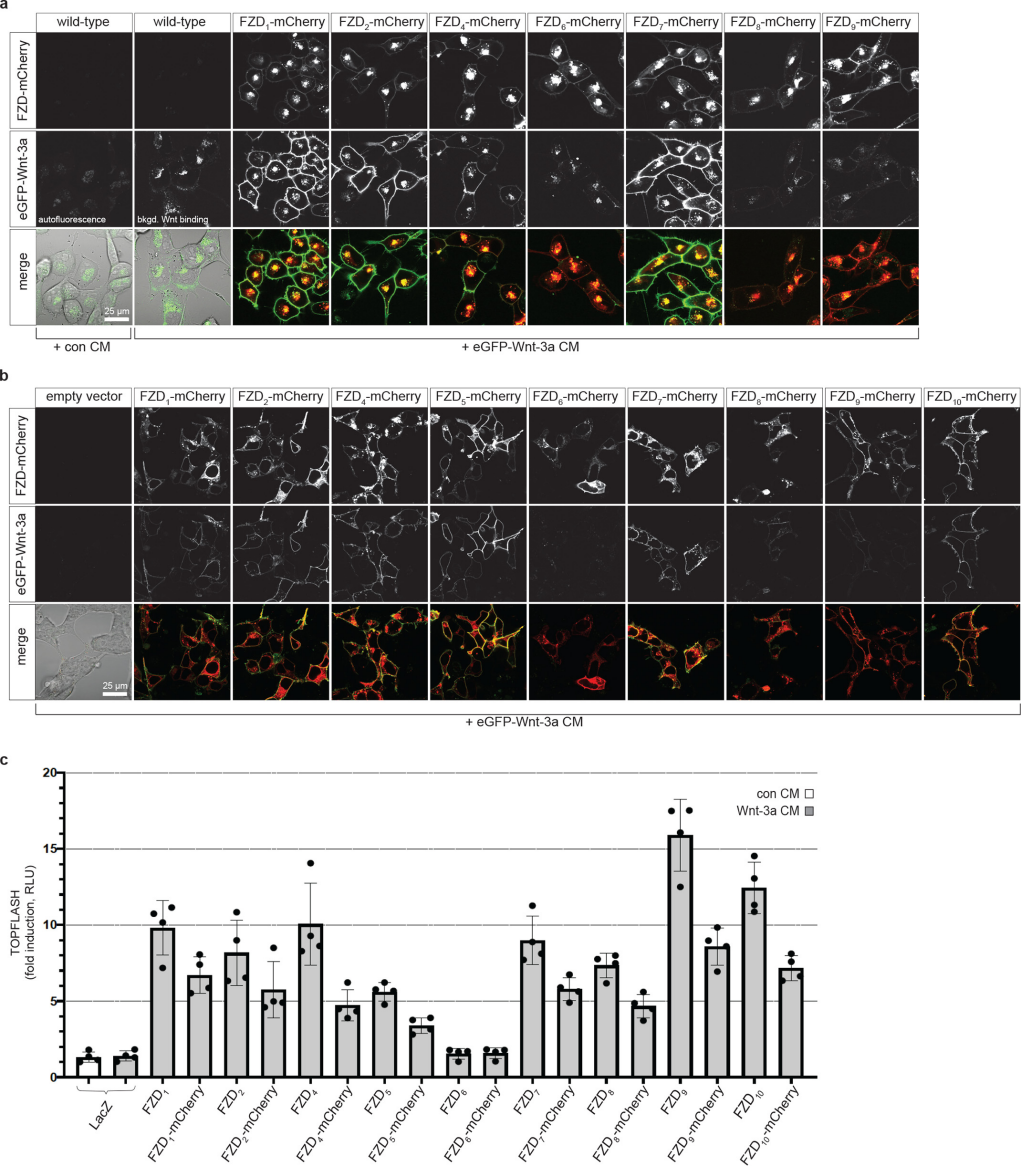
**Association of eGFP-Wnt-3a with different FZDs.** *a*, Laser scanning confocal microscopy images of living NCI-H1703 cells with stable integration of the indicated mouse FZD-mCherry gene, incubated for 3 h with eGFP-Wnt-3a conditioned medium (CM) derived from L cells. *b*, Laser scanning confocal microscopy images of living ΔFZD_1–10_^GFP-free^ HEK293 cells transiently transfected with the indicated mouse FZD-mCherry gene and incubated for 1 h with eGFP-Wnt-3a CM derived from L cells. A direct comparison of the fluorescent intensities between the two cell lines in *a* and *b* is provided in Fig. S3*b*. *c*, TOPFLASH TCF reporter assay responses in ΔFZD_1–10_ HEK293 cells, showing the relative Wnt/β-catenin signaling activity of the indicated mouse FZDs as well as their mCherry-tagged counterparts. Error bars represent ± S.D. from means of 4 independent biological samples, represented as solid dots. The experiment was performed 3 times with similar results.

Wnts bind to FZDs with some degree of promiscuity (9, 35), likely because of the conserved nature of the two Wnt-FZD interaction sites on the CRD (21, 22). To assess the relative interaction of eGFP-Wnt-3a to FZDs on live cells, we generated stable NCI-H1703 cell lines expressing moderate levels of FZD_1–10_ C-terminally tagged with mCherry at the plasma membrane (Fig. 4*a* and Fig. S3*b*). Cell lines with broadly similar expression levels of FZD_1,2,4,6,7,8,9_ at the cell surface were selected for imaging (Fig. 4*a*, *top*); however, suitable cell lines for FZD_3,5,10_ could not be obtained. The application of eGFP-Wnt-3a-containing CM resulted in a strong increase in membrane-associated green fluorescence when FZDs were expressed (Fig. 4*a*, *middle*). A low intensity of eGFP-Wnt-3a fluorescence was associated with WT NCI-H1703 cells (Fig. 4*a*, *middle*, *second from left*), presumably because of association with endogenous FZDs, LRP6, or other endogenously expressed Wnt receptors. We also used ΔFZD_1–10_ HEK293 (34) as host cells for transient transfection of the *FZD-mCherry* constructs. For this particular assay, we removed residual GFP expression from the parental cell lines using CRISPR/CAS9 to ensure that the eGFP signal solely originates from the addition of eGFP-Wnt-3a CM (Fig. S3*a*). A comparison of the relative FZD_1_-mCherry expression levels between stable NCI-H1703 cells and transiently transfected ΔFZD_1–10_^GFP-Free^ HEK293 cells, as well as their ability to associate with eGFP-Wnt-3a, is shown in Fig. S3*b*. The relative association of plasma membrane-localized green fluorescence (eGFP-Wnt-3a) with red fluorescence (mCherry-tagged FZDs) was interpreted as a relative measure of Wnt-FZD affinity. More variation in cell surface expression levels of the different FZD-mCherry constructs was observed in the ΔFZD_1–10_^GFP-free^ HEK293 cells than the NCI-H1703 cell lines (Fig. 4*b*, *top*). Nevertheless, similar differences were seen for relative cell surface association of eGFP-Wnt-3a in FZD-expressing cells, arguing for subtype selectivity of Wnt-3a binding to full-length FZDs (Fig. 4*b*, *middle*). Based on these imaging data, we approximate that eGFP-Wnt-3a associates more with cells expressing either FZD_1,_ FZD_2,_ FZD_4,_ FZD_5_, or FZD_7_ and less with cells expressing FZD_8_ or FZD_9_ (Fig. 4, *a* and *b*). Very low specific association of eGFP-Wnt-3a to FZD_6_-expressing cells was observed, which, similar to FZD_3_, shows strong preference for the Wnt/planar cell polarity signaling system (39). It must be underlined that it is a challenge to accurately quantify this association assay, given the differences in total and surface receptor expression of the FZD subtypes and limitations in distinguishing specific from unspecific binding, Thus, the relative binding of eGFP-Wnt-3a to different FZDs was approximated by visual inspection. The addition of mCherry to the C-terminal region of FZDs reduces, but does not prevent, their ability to transduce Wnt-3a signals when expressed in ΔFZD_1–10_ HEK293 cells (Fig. 4*c*). Interestingly, FZD_9_ maintains a strong Wnt-3a-induced signaling activity despite displaying relatively weak Wnt-3a cell surface association (Fig. 3, *a* and *b*) and displaying no significant activity in an earlier study using FZD_1,2,4,5,7,8_ null HEK293 cells (35). Taken together, these cellular association experiments suggest that full-length, soluble eGFP-Wnt-3a binds to a range of different full-length FZDs that are expressed on the surface of living cells. Nevertheless, this microscopic analysis provides merely an estimation of the relative ability of Wnt-3a to associate with different FZDs and cannot provide a thorough quantification of binding affinities.

### Quantification of eGFP-Wnt-3a binding to FZD_4_ in live cells

**Figure 5.**
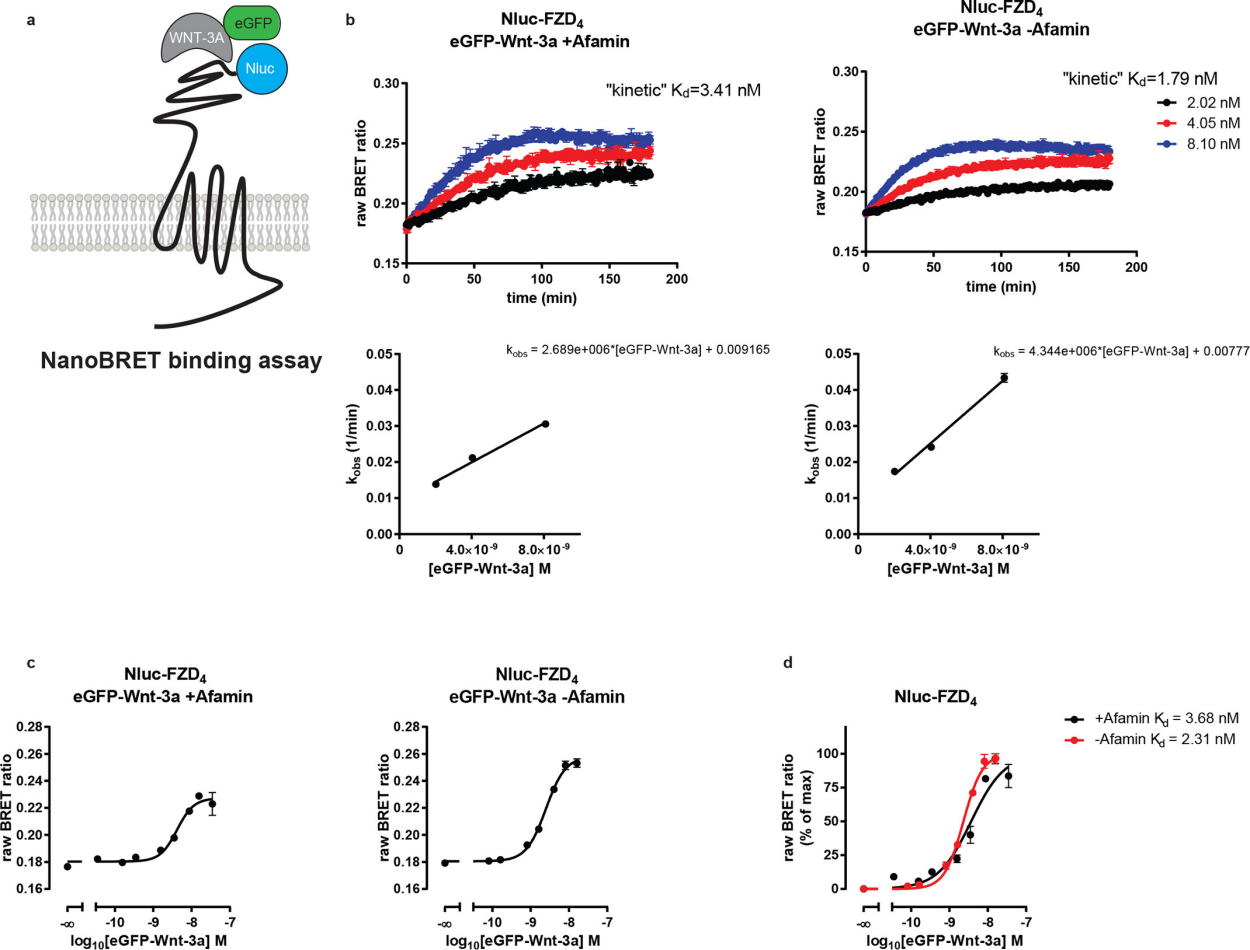
**eGFP-Wnt-3a binding to Nluc-FZD_4_.** *a*, Schematic illustration of the NanoBRET setup to detect eGFP-Wnt-3a binding to Nluc-FZD-tagged receptors. *b*, Association kinetics of the HEK293F suspension cell-derived eGFP-Wnt-3a to Nluc-FZD_4_ were determined by the detection of NanoBRET in transiently overexpressing living ΔFZD_1–10_ HEK293 cells over time. eGFP-Wnt-3a was produced either with (*left*) or without (*right*) cellular coexpression of Afamin. NanoBRET was sampled once per 60 s for 180 min. Data points are presented as means ± S.D. (+Afamin) or means ± S.E. (−Afamin) from *n* = 3 individual experiments, fitting a one-phase association model. The *k*_obs_ values from the individual eGFP-Wnt-3a association curves were plotted over eGFP-Wnt-3a concentration. *c*, Saturation curves are presented as sigmoidal curves with logarithmic eGFP-Wnt-3a concentrations (*left panel* for eGFP-Wnt-3a + Afamin and the *right panel* for eGFP-Wnt-3a −Afamin). Graphs present raw NanoBRET values obtained following 2-h ligand exposure to living ΔFZD_1–10_ HEK293 cells transiently overexpressing Nluc-FZD4. *d*, Raw BRET ratio values for eGFP-Wnt-3a binding from *c* were normalized to 0 and 100% to emphasize the difference in affinity. Data points are presented as mean ± S.E. from *n* = 3 individual experiments.

To more precisely quantify the binding of eGFP-Wnt-3a to FZDs, we employed a recently established NanoBRET assay (19, 40) that relies on an extracellularly localized Nanoluciferase (Nluc) on Nluc-FZD as the resonance energy donor and eGFP-Wnt-3a as the energy acceptor (Fig. 5*a*). We focused on FZD_4_ because (i) it presented as an intermediate binder of the eGFP-tagged Wnt-3a in the cellular assay shown in Fig. 4*a*, (ii) it is a well-described Wnt-3a receptor for mediating Wnt/β-catenin signaling (39), and (iii) it is well expressed on the cell surface. Nluc-FZD_4_ was transiently overexpressed in ΔFZD_1–10_ HEK293 cells (34), and eGFP-Wnt-3a was applied to the cells as conditioned medium. For NanoBRET binding assays, different eGFP-Wnt-3a preparations were used, from transiently transfected HEK293F suspension cells in defined serum-free medium, either with or without coexpressed Afamin (+Afamin and –Afamin, respectively), and from a stable L-cell line in normal serum-containing medium. The concentrations of eGFP-Wnt-3a were determined using GFP ELISAs (Fig. S4*a*). eGFP-Wnt-3a derived from HEK293F suspension cells associated relatively quickly (*k*_on_ = 2.69 × 10^6^ M^−1^·min^−1^ for +Afamin and 4.34 × 10^6^ M^−1^·min^−1^ for −Afamin) with Nluc-FZD_4_ and reached saturation after about 100 min (Fig. 5*b*). These kinetic binding assays provided *K_d_* values of 3.41 nm and 1.79 nm for the +Afamin and −Afamin preparations, respectively (Fig. 5*b*). In contrast, eGFP-Wnt-3a derived from L cells did not reach saturation even after 3 h of incubation, precluding an estimation of binding affinity from these kinetic experiments (Fig. S4*b*, *upper graph*).

The concentrations of eGFP-Wnt-3a used in the kinetic experiments were adjusted to the highest concentration possible of eGFP-Wnt-3a −Afamin and to the dilution factors of the assay format. Because of the lipophilicity of Wnts, high nonspecific binding can compromise the selective FZD-dependent signal, even though the NanoBRET-based binding assay provides an excellent signal-to-noise ratio. Therefore, we added Nluc-FZD_6_ to our NanoBRET assessment of eGFP-Wnt-3a binding to include a FZD that showed weak eGFP-Wnt-3a binding in the cellular association assay. In agreement with the lack of cell surface association to FZD_6_-mCherry-expressing cells (Fig. 4, *a* and *b*), no significant NanoBRET signal was detected between eGFP-Wnt-3a- and Nluc-FZD_6_-expressing cells (Fig. S4*c*). Likewise, the FZD-related Smoothened (SMO), which was used as a negative control because it does not associate with Wnt, failed to show significant binding in these assays (Fig. S4*c*). Cell surface expression of all Nluc-tagged FZD/SMO constructs was quantified using the detection of an N-terminal Nluc tag in an ELISA format (Fig. S4*d*).

To define a more precise binding affinity of Wnt-3a to FZD_4_, we incubated Nluc-FZD_4_-expressing cells with increasing concentrations of HEK293F cell- or L-cell-derived eGFP-Wnt-3a for 2 h (Fig. 5*c* and Fig. S4*b*). The raw BRET ratio representing eGFP-Wnt-3a to FZD_4_ binding increased in a concentration-dependent manner. For the HEK293F-derived eGFP-Wnt-3a, the affinity values were calculated to be the following: pK_d_ = 8.43 ± 0.06 (3.68 nm) for the +Afamin preparation and pK_d_ = 8.64 ± 0.02 (2.31 nm) for the −Afamin preparations (Fig. 5*c*). Similar to the kinetic experiments, the L-cell-derived eGFP-Wnt-3a binding did not reach saturation following 2 h of incubation, and the affinity can only be estimated (pK_d_ = 8.48 ± 0.05; 3.32 nm) (Fig. S4*b*, *lower*). Affinity values were determined from the normalized curves shown in Fig. 5*d* and Fig. S4*b*. Additionally, we performed NanoBiT binding assays (Fig. S4*e*) using HiBiT-tagged FZD_4_ and FZD_6_ to demonstrate that the lack of detectable eGFP-Wnt-3a binding to Nluc-FZD_6_ is not a consequence of steric hindrance caused by the Nluc tag (Fig. S4*f*). Finally, to exclude that agonist-induced receptor internalization compromises the assessments of ligand binding parameters, we performed bystander BRET-based live-cell internalization assays. Whereas β_2_-adrenergic receptors (β_2_AR-Nluc) expressed in ΔFZD_1–10_ HEK293 cells and stimulated with 10 μm isoprenaline were readily internalized, FZD_4_-Nluc did not internalize in response to 26.7 nm (1.0 µg/ml; Fig. S4*g*) of commercially available untagged humanWnt-3a.

## Discussion

Generally, FZDs show overlapping expression in tissues and cells (41), whereas sources of Wnt expression are more spatiotemporally restricted (41, 42), meaning Wnts are faced with considerable complexity regarding the engagement of FZDs to transduce Wnt signals. It remains unclear how cells achieve selectivity for particular Wnt-FZD pairs and what the underlying biochemical and biophysical properties are that determine receptor selectivity and pathway specification. Although coreceptors clearly afford some degree of specificity within the Wnt signaling network (34), much effort is focused on identifying the degree of specificity/redundancy for different Wnt-FZD combinations and to quantify their relative binding affinities. Recent work has provided a clearer overview of which Wnts functionally pair with which FZDs (9, 35), and although specificity indeed exists, a significant degree of promiscuity is apparent, in particular for Wnt-3a (9, 35). The availability of a soluble GFP-tagged version of Wnt-3a that functions similarly to WT Wnt-3a, together with new methodologies, such as the NanoBRET binding assay used here, allows quantification of Wnt-FZD binding in a precise, direct, and pharmacologically sound manner. As an initial step in this direction, we have focused on the precise quantification of the binding between Wnt-3a and FZD_4_. Employing real-time analysis on living cells, we have calculated the binding affinity of soluble Wnt-3a to cell surface-localized full-length FZD_4_ to be in the low-nanomolar range (2–3 nm) for the HEK293F cell-derived CM. Previous *in vitro* studies relying solely on the CRD of FZDs reported somewhat lower binding affinities for Wnt-3a-FZD_4_ (5.4 nm) (9). Thus, our work highlights the importance of studying ligand-receptor interactions under conditions that resemble native cellular environments, where contributions from potential cofactors/coreceptors, the glycocalyx, and membrane dynamics likely play an important role. Future efforts to study all Wnt-3a-FZD pairs under these native conditions, as well as additional Wnt-FZD pairs, will be a valuable contribution to our understanding of Wnt signaling selectivity.

The presented eGFP-Wnt-3a construct encodes a stable fusion protein that is secreted from cells in an EVI-dependent manner and transduces Wnt/β-catenin signaling in a variety of different biological contexts. Interestingly, there are some context-dependent differences in the relative activity of eGFP-Wnt-3a. There is little difference in the activity of WT Wnt-3a and eGFP-Wnt-3a when assayed in NCI-H1703 cells; however, there is an 80% reduction in activity using HEK293 cells. The reason for this discrepancy is unclear but likely reflects differences in gene expression profiles between the two cell lines. Indeed, when FZDs or LRP6 is overexpressed in HEK293 cells, the difference in activity between WT Wnt-3a and eGFP-Wnt-3a narrows significantly (Fig. 2*c* and Fig. S1*c*). Furthermore, TOPFLASH reporters are highly tuned cellular readouts for Wnt/β-catenin signaling, and when additional, arguably more physiologically relevant, readouts are used, such as endogenous LRP6 phosphorylation, Wnt-3a and eGFP-Wnt-3a display similar activities. Likewise, the equal ability of Wnt-3a and eGFP-Wnt-3a to promote axis duplication in *Xenopus laevis* embryos argues that fluorescent tagging of Wnt-3a at the N terminus does not appear to significantly compromise signaling activity within physiologically relevant contexts. We conclude that eGFP-Wnt-3a can be employed as a reliable tool to study Wnt signaling and Wnt-receptor interactions.

Structural studies describe the molecular mechanisms underlying the formation of Wnt-FZD complexes (21, 22) and allow some predictions to be made concerning Wnt-FZD-LRP6 ternary complex formation as well as oligomerization into higher-order structures (21, 22, 43–46), all of which should be relevant for the signaling activity and specificity of different Wnts. For binding of human Wnt-3 to the CRD of mouse FZD_8_ (21), which is of the most relevance to our study, some freedom of movement exists for Wnt tethered to the CRD by the “index finger-thumb clamping” mechanism. This positioning tolerance, or wobble, should allow Wnts some degree of flexibility in presenting their coreceptor interaction sites (43) and may help explain why, upon fusion of a 25-kDa GFP accessory protein, Wnt-3a retains its functional properties.

A soluble Flag-GFP-Wnt-3a fusion protein was recently shown to form 150-kDa complexes of GFP-Wnt-3a:Afamin and, to a lesser degree, higher-molecular-weight complexes, including a 200-kDa homotrimeric assembly of GFP-Wnt-3a (18). Considering the similarity of our eGFP-Wnt-3a, we assume a similar mixture of Afamin-bound and homomeric GFP-Wnt-3a complexes is present in our CM preparations. In apparent agreement with the findings that Wnt-3a can travel freely within the extracellular milieu (18), we also show that mCherry-Wnt-3a diffuses away from its cellular source and reaching cells within 3D spheroids that are located many cell diameters. In line with previous work (28), the coexpression of Afamin with eGFP-Wnt-3a in HEK293F suspension cells allows higher concentrations of eGFP-Wnt-3a to be obtained. Importantly, our NanoBRET data reveal different Wnt-3a-FZD_4_ binding characteristics for the two HEK293F cell CM preparations used (±Afamin), and it is tempting to speculate that differentially packaged Wnt and/or Wnt-Afamin complexes engage differently with FZDs, thereby altering their signaling specificity. Despite harboring concentrations of eGFP-Wnt-3a similar to those of HEK293F cell (−Afamin) preparations, L-cell-derived eGFP-Wnt-3a does not generate reliable binding assay data because of a lack of saturation binding. The L-cell-derived CM preparations should contain eGFP-Wnt-3a bound to Afamin (18, 28), because Afamin is present in the culture medium of L cells during production/secretion (because of its presence in serum). For HEK293F suspension cells (−Afamin), there is no serum present during production/secretion processes. However, serum is added at the final CM processing step to help stabilize Wnt-3a, so traces of Afamin will be present during the binding assays. Nevertheless, when Afamin is coexpressed with eGFP-Wnt-3a in HEK293F cells during the production/secretion process (+Afamin preparation), the binding affinity and kinetics are noticeably reduced compared with those of the −Afamin preparation. It is presently unclear whether Afamin can associate with Wnt proteins during the secretion process in coexpressing cells; however, it is conceivable that this influences the formation of Wnt complexes compared with that of cells that release “naked” Wnt into Afamin-containing extracellular environments. It will be interesting to study whether the formation of specific Wnt/Wnt-Afamin complexes guides distinct export routes, such as secretion, exosome association, or filipodial delivery to neighboring cells (47), and how packaging and carrier protein association affects FZD binding characteristics. Our paracrine signaling system, using spheroids of TOP-GFP reporter cells together with spheroids secreting fluorescently labeled Wnt proteins, should allow us to address some of these topics in more detail in the future.

The recently developed NanoBRET assay provides a high degree of selectivity because of the detection of specific energy transfer rather than measuring solely fluorescence associating with the membrane. This allowed the previous assessment of the binding of lipophilic BODIPY-cyclopamine to SMO (40) and FZD_6_ (48). Similarly, we exploit the assay's excellent signal-to-noise ratio here for the quantification of Wnt-FZD interaction. Whereas the assay allows kinetic analysis of ligand-receptor association, ligand binding follows very slow kinetics depending on the Wnt preparation. Irrespective of the Wnt preparation, the slow on-rate may be caused by predominant Wnt interactions with the extracellular matrix composed of, *e.g.* heparan sulfate proteoglycan such as glypicans (49). The apparent affinity of eGFP-Wnt-3a to full-length FZD_4_ in the low-nanomolar range, compared with previous data reporting affinities of Wnts for isolated CRDs, argues that the seven-transmembrane-spanning core contributes to ligand binding, even though the molecular details remain unresolved. Furthermore, it should also be pointed out that this binding assay does not provide any information on the stoichiometry of the ligand-receptor complex.

Our understanding of Wnt signaling has advanced considerably in the 25 years since the discovery of FZD (50) as the principal class of Wnt receptors. Some of the most notable milestones since this seminal discovery includes the discovery of Dickkopf (DKK) proteins as general inhibitors of Wnt/β-catenin (51), the discovery of LRP5/6 as Wnt/β-catenin pathway coreceptors (52–54), the discovery that Wnts undergo highly regulated secretion processes (55, 56), and the solving of the structural basis of Wnt-FZD binding (22). Throughout this period, however, there has been much difficulty associated with working with lipidated Wnt proteins, which has hampered progress in our understanding of ligand-receptor interaction specificity. The recent advances in generating fluorescently tagged versions of Wnt-3a proteins that retain functional properties (this work and a recent study [18]) provide an important new impetus in this direction. Here, we provide a clear example of the advances that can now be made with respect to unraveling the complexity of Wnt-FZD interactions in physiologically relevant environments.

## Materials and methods

### Plasmids

Mouse *pCS2*^+^*eGFP-Wnt-3a* was generated by inserting the NheI and XhoI restriction sites directly upstream of the *Wnt-3a* signal peptide. These restriction sites then were used to insert the *eGFP* ORF followed by a short linker (GGSGLE). For the cell line stably expressing *eGFP-Wnt-3a*, the ORF of the *eGFP-Wnt-3a* fusion was inserted between the NheI and NotI restriction sites of the *pEF1a-IRES-Neo* plasmid (a gift from Thomas Zwaka, Addgene, plasmid 28019) to create *pEF1a-eGFP-Wnt-3a*-*IRES-Neo*. *pCMV3-His-hAfamin* was obtained from Sino Biological (HG13231-CH).

*Mouse pmCherry-FZD_4_-mCherry* was constructed by replacing the eGFP ORF of *pEGFP-N2-FZD_4_-eGFP* (Addgene, plasmid 42197) with *mCherry* ORF amplified from *pCS2*^+^*LRP6-mCherry2* using the NEBuilder HiFi DNA assembly kit (New England Biolabs, E2621S). Mouse *pmCherry-FZD_1_*_,_*_2_*_,_*_5_*_,_*_6_*_,_*_7_*_,_*_8_*_,_*_9_*_,_*_10_-mCherry* constructs were prepared by replacing the *FZD_4_* ORF of *pmCherry-mFZD_4_-mCherry* with other *FZD* ORFs amplified from *PRK5-FZD_1_* (Addgene, 42253), *PRK5-FZD_2_* (Addgene, 42254), *PRK5-FZD_5_-1D4* (Addgene, 42267), *PRK5-FZD_6_* (Addgene, 42258), *PRK5-FZD_7_* (Addgene, 42259), *PRK5-FZD_8_* (Addgene, 42260), *PRK5-FZD_9_* (Addgene, 42261), and *PRK5-FZD_10_* (Addgene, 42262), respectively. The *pEF1a-IRES-Puro* plasmid for generating stably expressing FZD cell lines was assembled using NEBuilder by combining the NotI/ClaI linearized back bone of *pEF1a-IRES-NEO4* (Addgene, plasmid 28019) with the IRES from *pEF1a-IRES-NEO* and the Puro ORF from *Oct4-eGFP-PGK-PURO5* (Addgene, plasmid 31937). *FZD_1_*_,_*_2_*_,_*_4_*_,_*_6_*_,_*_7_*_,_*_8_*_,_*_9_*_,_*_10_-mCherry* ORFs then were excised from the corresponding *pmCherry-FZD-mCherry* plasmids and cloned in *pEF1a-IRES-Puro* using XhoI and NotI restriction sites. *7-TGP6* (Addgene, 24305) was used to generate fluorescent (7xTCF-GFP) reporter cells using a 3rd-generation lentiviral packaging system. *pVSV-G*, *pRSV-Rev*, and *pMDLg/pRRE* were gifts from Leonel Munoz.

Nluc-A_3_ was from Stephen Hill (University of Nottingham, UK) (19), and it was used as a template for cloning. The human FZD_4_ sequence (57) without its signal peptide (aa 1–36) was subcloned in-frame into an empty N-terminally tagged Nluc vector containing 5-HT_3_A signal peptide using BamHI and XbaI restriction sites with the following primers: 5′-GAC GGA TCC TTC GGG GAC GAG GAA GAG CGG-3′ (forward) and 5′-GTC TCT AGA TAC CAC AGT CTC ACT GCC TTT-3′ (reverse). To generate HiBiT-FZD_4_ and HiBiT-FZD_6_ constructs, Nluc sequences in Nluc-FZD_4_ and Nluc-FZD_6_ (48) were replaced with HiBiT sequences (nucleotides sequence, 5′-GTG AGC GGC TGG CGG CTG TTC AAG AAG ATT AGC-3′; amino acids sequence, VSGWRLFKKIS). The constructs were validated by sequencing (Eurofins GATC, Konstanz, Germany). For Nluc-FZD_4_, live-cell ELISA was used to quantify surface expression of the protein on cells. Nluc-SMO (coding for mouse Smoothened) and FZD_4_-Nluc were generated and characterized as described previously (37, 40). The synthetic construct anchoring two HaloTags, separated by a repetitive rigid linker, to the plasma membrane and C-terminally Nluc-tagged β_2_AR (β_2_AR-Nluc) were kindly provided by Jan Möller and Martin J. Lohse (Max-Delbrueck Center for Molecular Medicine, Berlin, Germany).

### Cell culture

Human embryonic kidney 293T (HEK293T) cells (DSMZ ACC-635), ΔFZD_1–10_ HEK293 cells (34) mouse fibroblast L-cells (ATCC^®^ CRL-2648^TM^), stably expressing Wnt-3a L cells (ATCC^®^ CRL-2647^TM^), and stably expressing eGFP-Wnt-3a L cells (clone SF75, generated in this project) were cultured in Dulbecco's modified Eagle medium (Thermo Fisher, Waltham, MA) supplemented with 10% fetal bovine serum (Thermo Fisher) and 1% penicillin-streptomycin (P/S; Gibco; Thermo Fisher). NCI-H1703 human lung carcinoma cells (ATCC^®^ CRL-5889^TM^) and NCI-H1703 cell lines with stable integration of mouse FZD_1,2,4,6,7,8,9_-mCherry (generated in this project) were cultured in Roswell Park Memorial Institute (RPMI) 1640 medium (Thermo Fisher) supplemented with 10% FBS and 1 mm sodium pyruvate (Thermo Fisher). All cell lines were maintained at 37 °C and 5% CO_2_. Expi293F^TM^ suspension cells (Thermo Fisher, A14527) were cultured in Expi293^TM^ expression medium (Thermo Fisher) at 37 °C and 8% CO_2_ with 125 rpm orbital shaking in a New Brunswick S41i CO_2_ shaking incubator (Eppendorf). Cell densities and viability were determined using a Countess II automated cell counter (Life Technologies).

### Preparation of wnt-3a and eGFP-Wnt-3a CM

For L-cell CM, Wnt-3a CM was prepared from mouse L cells stably transfected with mouse Wnt-3a (ATCC^®^ CRL-2647^TM^). eGFP-Wnt-3a CM was prepared from a stable L-cell line generated from mouse L cells (ATCC^®^ CRL-2648^TM^) stably transfected with eGFP-Wnt-3a (clone SF75). Control CM was prepared from nontransfected L cells (ATCC^®^ CRL-2648^TM^). Cells were maintained at 37 °C and 5% CO_2_.

For the +Afamin HEK293F^TM^ CM, suspension cells growing in Expi293^TM^ expression medium (60 ml, 2.5 × 10^6^ cells/ml) were cotransfected with 10 µg of either *pCS2*^+^-*Wnt-3a* or *pCS2*^+^-*eGFP-Wnt-3a* together with 50 µg of *pCMV-His-Afamin* plasmid using ScreenFect^®^ UP-293 (ScreenFect GmbH) according to the manufacturer's instructions. For the −Afamin HEK293F^TM^ CM, 40 µg of *pCS2*^+^-*eGFP-Wnt-3a* together with 20 µg of *pCMV* plasmid. Wnt-3a and eGFP-Wnt-3a CM were collected 96 h posttransfection. The corresponding control CM was generated from cells transfected with *pCMV* plasmid.

For downstream experiments, the serum-containing CM samples from L cells were first centrifuged at 130 relative centrifugal force for 10 min to remove cells and then at 3,000 relative centrifugal force for 30 min to remove any remaining cellular debris and insoluble material. This “raw” CM then was concentrated 13-fold using Vivaspin turbo 15 and 30,000-molecular-weight-cutoff ultra filters (Satorius AG, Göttingen, Germany) and exchanged to the desired cell culture medium using Sephadex G-25 PD10 desalting columns (GE Healthcare Bio-Science, Freiburg, Germany). Serum-free CM from HEK 293F^TM^ suspension cells were prepared as for L-cell CM but concentrated only 5-fold using Vivaspin turbo 15 and 30,000-molecular-weight-cutoff ultra filters. The final concentration and integrity of eGFP-Wnt-3a in the CM samples were determined using ELISA (GFP ELISA^®^ kit, Abcam, ab171581).

### WB analysis

For Western blot (WB) analysis of Wnt-3a and eGFP-Wnt-3a, proteins were first purified from serum-containing CM using Cibacron blue 3G coupled to Sepharose 6 Fast Flow (Blue-Sepharose 6 Fast Flow, GE Healthcare). Conditioned medium was supplemented with Triton X-100 to a final concentration of 1%. Blue-Sepharose beads were washed three times with Blue-Sepharose buffer (BS-buffer) (150 mm KCl, 50 mm Tris-HCl, pH 7.5, 1% Triton X-100) containing Complete^®^ protease inhibitor mixture (Roche, Basel, Switzerland). Washed beads were added to the samples and rotated overnight at 4 °C. The next day, beads were washed three times in BS-buffer and proteins eluted by heating in Laemmli sample buffer.

For Western blot analysis of endogenous LRP6, HEK293T as well as NCI-H1703 cells cultured in 24-well plates were treated with 150 μl control, Wnt-3a, or eGFP-Wnt-3a CM for 90 or 120 min. Cells were lysed in 1% Triton lysis buffer (1% Triton X-100, 50 mm Tris-HCl [pH 7.0], 150 mm NaCl, 25 mm NaF, 5 mm Na_3_VO_4_, 0,1% NP-40, 1 mm EDTA) containing Complete^®^ protease inhibitor. After 15 min of incubation on ice, cell lysates were centrifuged at 10,000 × *g* at 4 °C for 10 min, and lysates were supplemented with Laemmli sample buffer and heat denatured.

Samples were separated by SDS-PAGE before transfer to a PVDF membrane using a Bio-Rad Transblot-Turbo system (Bio-Rad). Membranes were blocked at room temperature for 1 h in 5% BSA–TBST blocking buffer (5% BSA, 137 mm NaCl, 2.7 mm KCl, 19 mm Tris base [pH 7.4], 0.1% Tween-20) and transferred to a BioLane HTI automated Western blotting processor for antibody incubation and washing steps. The following antibodies were used: anti-Wnt-3a (ab28472, 1:1000, Abcam), anti-LRP6 ([1C10], 1:1,000, Abcam), anti-phospho-LRP6 (Sp1490, 1:1,000, Cell Signaling), anti-GFP (ab1828, 1:2,000, Abcam), anti-Vinculin (E1E9V, 1:1,000, Cell Signaling), and HRP-conjugated anti-rabbit or anti-mouse secondary antibodies (Dako). For semiquantitative detection of protein bands, the membranes were incubated with ECL Prime (GE-Healthcare Bioscience) and imaged using a ChemiDoc^TM^ touch imaging system (Bio-Rad).

For Western blots shown in Fig. 2*a*, Blue-Sepharose pulldown of Wnt-3a was performed as described previously (58). In brief, 1.0 × 10^6^ HEK293T and HEK293T EVI KO2.9 (58) cells were seeded into 6-well plates. Twenty-four hours later, cells were transfected with 500 ng pcDNA, Wnt-3a, or eGFP-Wnt-3a and 1.5 µl TransIT (Mirus, VWR) per well according to the manufacturer's instructions. Twenty-four hours after transfection, supernatants were recovered and centrifuged at 2,000 × *g* for 10 min to remove cell debris. Remaining cells were harvested and lysed in BS-buffer (150 mm KCl, 50 mm Tris-HCl, pH 7.5, 1% [v/v] Triton X‐100, protease inhibitors) and 6× Laemmli buffer. Supernatants were supplemented with Triton X‐100 to a final concentration of 1% (v/v), and prewashed Blue-Sepharose 6 Fast Flow (17‐0948‐01, GE Healthcare) was added. After overnight rotation at 4 °C, samples were washed several times with BS buffer and centrifuged at 2,700 × *g* for 5 min at 4 °C. The beads were resuspended in 2× Laemmli buffer. Supernatants and cell lysates were incubated at 96 °C for 5 min and then subjected to SDS gel electrophoresis and Western blot analysis. The following antibodies were used: anti-EVI antibody (1:500, monoclonal mouse, clone YJ5, BioLegend, 655902), anti-HSC70 (1:1,000, mouse, Santa Cruz, sc‐7298), anti-Wnt-3A (1:1,000, rabbit, Abcam, ab28472), and secondary HRP-coupled goat anti‐mouse (1:10,000, Jackson ImmunoResearch) and goat anti‐rabbit (1:10,000, Jackson ImmunoResearch).

### Confocal laser scanning microscopy

For the microscopy analysis of eGFP-Wnt-3a binding to Frizzled receptors, ΔFZD_1–10_^GFP-Free^ HEK293 cells were transfected with 5 ng of *pCherry-mFzd-mCherry* and 220 ng empty *pCS2*^+^ in µ-Slide 8-well chambers (Ibidi, catalog no. 80826), and, as a control, ΔFZD_1–10_^GFP-Free^ HEK293 cells were transfected with 225 ng empty *pCS2*^+^. Twenty-four hours posttransfection, control or eGFP-Wnt-3a CM was added to cells and incubated for 1 h. For microscopy, cell medium was exchanged for Gibco FluoroBrite DMEM (Thermo Fisher, Waltham, MA) supplemented with 10% FBS, 2 mm l-glutamine (Gibco, Thermo Fischer), and 1% P/S. For analysis of eGFP-Wnt-3a binding to WT NCI-H1703 cells or the NCI-H1703 cell lines with stable integration of the *mFZD-mCherry* genes, cells were seeded in µ-Slide 8-well chambers so that they were 80% confluent for microscopy after 2 days. Prior to imaging, cells were treated with control or eGFP-Wnt-3a CM for 3 h. For microscopy, the cell culture medium was replaced with phenol red-free RPMI mixed 1:1 with FluoroBrite, supplemented with 10% FBS, 1 mm sodium pyruvate, 2 mm l-glutamine, and 1% P/S.

Image acquisition was performed using a Zeiss LSM 800 microscope (Zeiss, Jena, Germany) fitted with a 63×/1.4 oil differential interference contrast (UV) VIS-IR Plan-Apochromat objective (Zeiss, Jena, Germany) and a GaAsP-PMT detector. The red mCherry and green eGFP fluorescent proteins were excited at 561 nm and 488 nm, respectively, and their respective emissions were captured in the range of 570–700 nm and 400–576 nm, employing the standard filter sets where appropriate. Images were analyzed using Fiji (59).

### CRISPR/Cas9 knockout

ΔFZD_1–10_ HEK293T cells were generated as described previously using pSPCas9(BB)-2A-GFP (PX458) (34). However, these cells display significant background cytosolic GFP fluorescence that interferes with imaging, so we removed the randomly integrated *GFP* from the genome of these cells using CRISPR/Cas9 editing. We used the crispor online tool (crispor.trefor.net) (60) to identify two 20-bp protospacers, one at the 5′ start (5′-GCTCCTCGCCCTTGCTCACT-3′, gRNA51) and one in the middle (5′-GCAACTACAAGACCCGCGCCG-3′, gRNA449) of the *GFP* ORF. These were used simultaneously to remove ∼335 bp at the 5′ end of the *GFP* ORF. Each of the complementary DNA fragments was designed with the necessary overhangs, annealed, and ligated into the BbsI restriction site of the pX330-U6-Chimeric_BB-CBh-hSpCas9 (D10) vector (61), generating two plasmids encoding a single guide RNA, each harboring a *GFP-*specific 20-bp protospacer under the control of a U6 RNA polymerase III promoter.

ΔFZD_1–10_ HEK293T cells cultured in 6-well plates were transfected with 1 µg pX330-U6-gRNA51-CBh-hSpCas9 (D10) and 1 µg X330-U6-gRNA449-CBh-hSpCas9 (D10) using ScreenFect^®^ A (ScreenFect GmbH) according to the manufacturer's 1-step protocol. Twenty-four hours posttransfection, the medium was exchanged. Forty-eight hours posttransfection, single cells were transferred to 96-well plates using limited dilutions to amplify clonal lines. Cells were screened using fluorescence microscopy for GFP-negative clonal lines. The new ΔFZD_1–10_ HEK293T clonal GFP knockout line was named ΔFZD_1–10_^GFP-Free^ HEK293T to differentiate it from the parental ΔFZD_1–10_ HEK293T cells that express cytosolic GFP (34).

### Xenopus laevis axis duplication assays

*In vitro* fertilization, embryo culture, preparation of mRNA, and microinjection were carried out as described (62). mRNA was prepared using the Invitrogen^TM^ mMESSAGE mMACHINE^TM^ SP6 transcription kit according to the manufacturer's protocol. *Xenopus laevis* embryos were injected equatorially with 2.5 pg of either *Wnt-3a*, *eGFP-Wnt-3a*, or *lacZ* mRNA into each of the two ventral blastomeres at the four-cell stage. Embryos were left to develop at 18 °C until stage 28 for analysis. The *Xenopus* work has been approved by the state review board of Baden-Wuerttemberg, Germany (license no. G-13/186 and G-141/18) and performed according to federal and institutional guidelines.

### Generation of cell lines stably expressing eGFP-Wnt-3A and FZD-mCherry

L-cells were transfected with 2 µg *pEF1a-eGFP-Wnt3a-IRES-Neo*, and NCI-H1703 cells were transfected with 2 µg *pEF1a-FZD-mCherry-IRES-Neo* in 6-well plates using ScreenFect^®^ A (ScreenFect GmbH) according to the manufacturer's 1-step protocol. Twenty-four hours posttransfection, the medium was exchanged, and 48 h posttransfection one-tenth of the cells were transferred to 10-cm^2^ dishes and cultured in their respective cell culture medium supplemented with 1.2 mg/ml (L cells) or 700 µg/ml (NCI-H1703) G418 (Sigma-Aldrich). Selection was performed for 12 days. Clonal lines were obtained by limited dilutions in 96-well plates, and, for analysis in this study, cell lines expressing similar levels of FZD-mCherry on the plasma membrane were selected by microscopy screening.

### Generation of 7-TGP fluorescent TOP-GFP reporter cell line

For virus packaging, 10-cm dishes of HEK 293T cells at 70% confluence were transfected with 10 µg of *7-TGP*, 2.8 µg of *pVSV-G*, 2.5 µg of *pRSV-Rev*, and 5 µg *pMDLg/pRRE* using ScreenFect^®^ A (ScreenFect GmbH) by following the manufacturer's instructions. Twenty-four hours posttransfection, cell culture supernatants were collected in a 10-ml syringe, filtered through a 0.45-μm filter, and directly added to HEK293T target cells. Target cells were incubated with virus-containing medium overnight. A second round of lentivirus harvest/lentivirus infection was performed as described above on the same HEK 293T target cells. HEK 293T target cells then were allowed to recover in fresh DMEM for 24 h before selection in puromycin-containing medium for 72 h. Surviving cells were seeded at 0.5 cells/well in 5 96-well plates for single-colony amplification. After 2 weeks, single colonies were picked and split between two 96-well plates on separate plates and treated overnight with either control or Wnt-3a CM, and clones with the best Wnt-induced signal and lowest background were selected using fluorescence microscopy.

### TOPFLASH reporter assays

To test the biological activity of eGFP-Wnt-3a, 6 × 10^4^ HEK293T or 4.5 × 10^4^ NCI-H1703 cells cultured in 96-well plates were transfected with 20 ng TCF firefly luciferase (TOPFLASH), 2 ng CMV Renilla luciferase, and 78 ng *LacZ* using ScreenFect^®^ A according to the manufacturer's 1-step protocol (ScreenFect GmbH). Twenty-four hours posttransfection, medium was replaced by control, Wnt-3a, or eGFP-Wnt-3a CM, and cells were incubated for another 24 h before harvesting cell lysates.

For the TOPFLASH luciferase assay shown in Fig. 2*c*, 4.0 × 10^4^ HEK293T and HEK293T FZD knockout cells (HEK293T ΔFZD_1,2,4,5,7,8_ clone1 [35]) were seeded per well in 96-well plates. Twenty-four hours after seeding, cells were transfected with 25 ng TCF4/Wnt-Firefly luciferase (63), 5 ng CMV-Renilla luciferase, with or without 2 ng pCMV-XL4 FZD2 (Origene, SC127603), 2 ng pcDNA, Wnt-3a, or eGFP-Wnt-3a, and 0.1 µl TransIT-LT1 (Mirus, VWR) per well according to the manufacturer's instructions. Dual-luciferase readout was performed using the Mithras LB940 plate reader (Berthold Technologies) 24 h later. TCF4-FLUC values were normalized to the CMV-RLUC values.

To test the biological activity of the different FZDs and their mCherry-tagged counterparts, 6 × 10^4^ ΔFZD_1–10_^GFP-free^ HEK293 cells in 96-well plates were transfected with TOPFLASH/Renilla reporter plasmids (20/2 ng), 0.2 ng of either *pRK5-FZD* or *pCherry-FZD-mCherry*, and 77 ng *lacZ*. Twenty-four hours posttransfection, culture medium was replaced with either control or Wnt-3a CM, and cells were incubated for another 24 h before harvesting cell lysates for luciferase assays. For the luciferase assays, cells from 96-well plates were harvested in 43 µl passive lysis buffer (Promega, Mannheim, Germany) and processed according to the manufacturer's protocol. TOPFLASH luciferase values were normalized to control Renilla luciferase values. All error bars shown are standard deviations from the mean (±S.D.) of the indicated number of independent biological samples within an experiment, and all experiments were performed at least 3 times, unless indicated otherwise in the figure legends. Data were analyzed using GraphPad Prism 8.

### Exosome purification and analysis

HEK293T cells were maintained in DMEM (Gibco) supplemented with 10% fetal calf serum (Biochrom) at 37 °C in a humidified atmosphere with 5% CO_2_. Cells were transiently transfected with plasmids using Screenfect^®^ A (Screenfect GmbH) according to the manufacturer's instructions. Extracellular vesicles were purified by differential centrifugation as described previously (64, 65). In short, supernatants from mammalian cells were subjected to sequential centrifugation steps of 750 × *g*, 1,500 × *g*, and 14,000 × *g* before pelleting exosomes at 100,000 × *g* in a Beckman Coulter TLA 100.3 rotor. The exosome pellets were washed at 100,000 × *g* for 1 h. The supernatant was discarded, and exosomes were dissolved in 1/100 their original volume in 1× PBS. Isolation of exosomes by magnetic-activated cell sorting involved positive selection using MicroBeads recognizing the tetraspanin protein CD9, CD63, or CD81. The immunobead isolation was performed using the exosome isolation kit Pan-130-110-912 according to the manufacturer's protocol (Miltenyi Biotec GmbH). First, EVs are magnetically labeled during a short incubation period and then loaded onto a µ column, which is placed in the magnetic field of a µMACS^TM^ separator. The magnetically labeled EVs are retained within the column. After removing the column from the magnetic field, the intact EVs are collected by elution with isolation buffer and lysed with radioimmunoprecipitation assay buffer. The exosome samples were further analyzed by Western blotting. Antibodies were used against Wnt-3a, 1:500 (WB; rabbit, Abcam), Syntenin, 1:2,000 (WB; rabbit, Abcam), and Alix, 1:2,000 (WB; mouse, Santa Cruz Biotechnology, 1A12).

### 3D spheroid fusion assays

Superhydrophobic-superhydrophilic patterned glass slides (7.5 by 2.5 cm) were obtained from Aquarray GmbH, Germany. Each slide has three arrays, and each array has 14 by 14 hydrophilic spots (1 mm by 1 mm). Two hundred cells (200 nl of 1 × 10^6^ cells/ml) from either the mCherry-Wnt-3a-expressing, control LacZ-expressing, or TOP-GFP reporter lines were seeded into individual spots on the patterned slides in defined patterns using an I-DOT dispenser (Dispendix GmbH, Germany). After culturing as hanging droplets for 2 days to allow HEK293 cell spheroid formation, neighboring spots were merged by adding 1 μl DMEM to each spot (*e.g.* A1 and A2). The resulting fused spheroids were then analyzed by fluorescence microscopy (Zeiss LSM 800 confocal fluorescent microscope) 24 h and 48 h after merging.

### NanoBRET binding assay

ΔFZD_1–10_ HEK293 cells (34) were transiently transfected in suspension using Lipofectamine^®^ 2000 (Thermo Fisher Scientific). A total of 4 × 10^5^ cells were transfected in 1 ml with 10 ng of Nluc-tagged receptor plasmid DNA or 100 ng of HiBiT-tagged receptor plasmid DNA and 990 or 900 ng of pcDNA plasmid DNA. The cells (100 µl) were seeded onto a poly-d-lysine-coated black 96-well cell culture plate with a solid flat bottom (Greiner BioOne). Twenty-four hours posttransfection, the cells were washed once with 200 µl of Hanks' balanced salt solution (HBSS; HyClone). In the kinetics experiments, the cells were preincubated with 50 µl of the Nluc substrate vivazine (1:50 dilution; Promega) in a complete, non-phenol red DMEM (HyClone) supplemented with 10 mm HEPES for 1 h at 37 °C without CO_2_. Subsequently, 50 µl of eGFP-Wnt-3a conditioned medium or control medium supplemented with 10 mm HEPES was added, and the BRET signal was measured every 60 s for 180 min at 37 °C (181 measurements, no CO_2_). In the saturation-binding experiments, the cells were incubated with different concentrations of eGFP-Wnt-3a conditioned medium (90 µl) supplemented with 10 mm HEPES for 120 min at 37 °C with no CO_2_. Next, for the Nluc-tagged constructs, 10 µl of the Nluc substrate furimazine was added (1:100 dilution; Promega), and for the HiBiT-tagged constructs, 90 μl of a mix of furimazine (1:100 dilution; Promega) and LgBiT (1:200 dilution; Promega) was added. The cells were incubated for another 10 min prior to the BRET measurements. The BRET ratio was determined as the ratio of light emitted by eGFP (energy acceptor) and light emitted by Nluc/HiBiT-tagged receptors (energy donors). The BRET acceptor (bandpass filter, 535–30 nm) and BRET donor (bandpass filter, 475–30 nm) emission signals were measured using a CLARIOstar microplate reader (BMG). eGFP fluorescence was measured prior to reading BRET (excitation, 470–15 nm; emission, 515–20 nm). Data were analyzed using GraphPad Prism 6.

### Bystander BRET receptor internalization assay

Parental and ΔFZD_1–10_ HEK293 cells were transiently transfected in suspension using Lipofectamine^®^ 2000. 200 ng of β_2_AR-Nluc or FZD_4_-Nluc plasmid DNA was added along with 800 ng DNA of the membrane-anchored HaloTag construct to a cell suspension of 4 × 10^5^ cells per ml. One hundred microliters of cell suspension was plated in white 96-well cell culture plates with solid flat bottoms (Greiner BioOne). Twenty-four hours after transfection, HaloTag^®^ NanoBRET^TM^ 618 ligand (Promega) was added to each well to a final concentration of 50 nm. Forty-eight hours posttransfection, cells were washed with 100 µl HBSS incubated in 90 µl Nluc substrate furimazine in HBSS (1:1,000 dilution; Promega) for 2 to 5 min. Subsequently, baseline BRET was recorded in three consecutive reads. Next, 10 µl of vehicle control or 10-fold ligand, recombinant human Wnt-3a (RnD) or isoprenaline (Sigma), solution was added, and BRET was recorded for an additional 2 h. BRET measurements were conducted at 37 °C using a CLARIOstar microplate reader (BMG) equipped with monochromators to separate light emitted by C-terminally Nluc-tagged receptors (420–480 nm; energy donor) and the membrane-anchored red fluorescent HaloTag^®^ NanoBRET^TM^ 618 ligand (600–660 nm; energy acceptor). The cycle time was set to 2 min with an integration time of 0.3 s. BRET ratio was defined as emission intensity of HaloTag^®^ NanoBRET^TM^ 618 Ligand over Nluc and analyzed using GraphPad Prism 6.

### Live-cell ELISA

For quantification of cell surface receptor expression by labeling with anti-Nluc antibody, ΔFZD_1–10_ HEK293 cells at a density of 4 × 10^5^ cells/ml were transfected in suspension using Lipofectamine^®^ 2000 with 10 ng of the indicated receptor plasmid DNA and 990 ng of pcDNA plasmid DNA. The cells (100 µl) were seeded onto a PDL-coated transparent 96-well plate with a flat bottom and grown overnight. Twenty-four hours later, the cells were washed once with 0.5% BSA in PBS and incubated with a mouse anti-Nluc (2 µg/ml, MAB10026; RnD Systems) in 1% BSA–PBS for 1 h at 4 °C. Following incubation, the cells were washed three times with 0.5% BSA–PBS and incubated with a horseradish peroxidase-conjugated goat anti-mouse antibody (1:3,000, no. 31430; Thermo Fisher Scientific) in 1% BSA–PBS for 1 h at 4 °C. The cells were washed three times with 0.5% BSA/PBS, and 50 µl of the peroxidase substrate 3,3′,5,5′-tetramethylbenzidine (T8665; Sigma-Aldrich) was added. The cells were incubated further for 20 min, and upon development of a blue product, 50 µl of 2 M HCl was added and the absorbance was read at 450 nm using a BMG Ω POLARstar plate reader. The data were analyzed in GraphPad Prism 6.

### Data and statistical analysis

For the TOPFLASH reporter assays shown in Fig. 1*b*, 3*a*, and 4*c*, as well as Fig. S1*d* and Fig. S4*a*, error bars show standard deviation from the means (±S.D.) of *n* = 4 independent biological samples within an experiment, and all experiments were performed at least 3 times, unless indicated otherwise. For the TOPFLASH assay in Fig. 2*c*, the error bars show standard deviations from the means (± S.D.) from *n* = 5 independent biological samples. The experiment was performed twice with similar results.

For the NanoBRET binding data presented, the eGFP-Wnt-3a saturation binding curves were fitted using a three- or four-parameter nonlinear regression model. The binding curves represent mean ± S.E. of the mean from three independent experiments, each performed in two technical replicates. Affinity values are presented as a best-fit *K_d_* ± S.D. A one-phase association model was used to analyze eGFP-Wnt-3a binding kinetics data:

$$Y=Y_{0}+\left(plateau-Y_{0}\right)\times (1-e^{\left(-k_{obs}*x\right)})$$

NanoBRET binding models were selected based on an extra-sum-of-square F-test (*p* < 0.05). NanoBRET binding data for Nluc-FZD_6_, Nluc-SMO, and Nluc-FZD_4_ (Fig. S5) were analyzed for differences with unpaired *t* tests to compare raw BRET ratios of eGFP-Wnt-3a medium binding with control medium binding for each construct.

Live-cell ELISA data were analyzed using GraphPad Prism 6 and represent means ± S.E. of three individual experiments (biological replicates) performed in triplicates (technical replicates). Live-cell ELISA data were analyzed for differences with one-way analysis of variance with Fisher's least significant difference post hoc analysis. Significance levels (*p* < 0.0001) are indicated by asterisks or hashtags.

## Data availability

Data supporting the findings of this manuscript are available from the corresponding authors upon reasonable request.

## Supplementary Material

Supporting Information
