# Supplementary material for: eGFP-tagged Wnt-3a enables functional analysis of Wnt trafficking and signaling and kinetic assessment of Wnt binding to full-length Frizzled
Source: J Biol Chem. 2020 May 7;295(26):8759–74. doi: 10.1074/jbc.RA120.012892 (PMC7324525; doi:10.1074/jbc.RA120.012892)
Supplement: Supporting Information [file supp_295_26_8759__index.html]

eGFP-tagged Wnt-3a enables functional analysis of Wnt trafficking and signaling and kinetic assessment of Wnt binding to full-length Frizzled — eGFP-Wnt-3a selectively binds Frizzleds — eGFP-tagged Wnt-3a enables functional analysis of Wnt trafficking and signaling and kinetic assessment of Wnt binding to full-length Frizzled — eGFP-Wnt-3a selectively binds Frizzleds — Supporting Information 

# eGFP-tagged Wnt-3a enables functional analysis of Wnt trafficking and signaling and kinetic assessment of Wnt binding to full-length Frizzled

## Supporting Information

- Supporting Information (to be published online) - Supporting Figures S1, S2, S3, S4
