# Supplementary material for: eGFP-tagged Wnt-3a enables functional analysis of Wnt trafficking and signaling and kinetic assessment of Wnt binding to full-length Frizzled
Source: J Biol Chem. 2020 May 7;295(26):8759–74. doi: 10.1074/jbc.RA120.012892 (PMC7324525; doi:10.1074/jbc.RA120.012892)
Supplement: Supporting Information [file supp_RA120.012892_158599_3_supp_525429_q9wjy5.pdf]

Supplementary Information For:

Engineered eGFP-tagged Wnt-3a allows functional analysis of Wnt trafficking, signaling and kinetic assessment of Wnt binding to full length Frizzled

Janine Wesslowski[1#], Pawel Kozielewicz[2#], Xianxian Wang[1], Haijun Cui[1], Hannes Schihada[2], Dominique Kranz[3], Pradhipa Karuna[4], Pavel Levkin[1], Julia Gross[4], Michael Boutros[3] Gunnar Schulte[2*] and Gary Davidson[1*]

From the [1] Institute of Biological and Chemical Systems – Functional Molecular Systems (IBCS-FMS), Karlsruhe Institute of Technology (KIT), Karlsruhe, Germany; [2] Section of Receptor Biology & Signaling, Dept. Physiology & Pharmacology, Karolinska Institutet, S-171 65, Stockholm, Sweden; [3] Division of Signaling and Functional Genomics, German Cancer Research Center (DKFZ) and Heidelberg University, Heidelberg, Germany; [4] Hematology and Oncology/Developmental Biochemistry, University Medical Center Goettingen, Goettingen, Germany.

Running title: *eGFP-Wnt-3a selectively binds Frizzleds*

[#] These authors contributed equally to this work: Janine Wesslowski and Pawel Kozielewicz

[*] To whom correspondence should be addressed:
Gary Davidson, Institute of Biological and Chemical Systems – Functional Molecular Systems (IBCS-FMS) Karlsruhe Institute of Technology (KIT), Karlsruhe, Germany; gary.davidson@kit.edu; Tel. +49-721-608-26103

Gunnar Schulte, Section of Receptor Biology & Signaling, Dept. Physiology & Pharmacology, Karolinska Institutet, S-171 65, Stockholm, Sweden; gunnar.schulte@ki.se; Tel. +46-8-52487933

Keywords: Wnt signaling, GPCR, BRET, fusion protein, membrane protein, trafficking, Frizzled, ligand binding, NanoBRET, NanoBiT

a

MAPLGYLLVLCSLKQALG-AS-EGFP-GGSGLE-WNT3A

mWnt3a signal sequence (SS), Restriction site: NheI (AS), EGFP ORF (- stop), Linker, including *XhoI* site (*LE*), mWnt3a ORF (- SS)

Nucleotide sequence:

ATGGCTCCTCTCGGATACCTCTTAGTGCTCTGCAGCCTGAAGCAGGCTCTGGGCGCTAGC**ATG**GTGAGCAAGGGCGAGGAGCTGTTCACC
GGGGTGGTGCCCATCCTGGTCGAGCTGGACGGCGACGTAAACGGCCACAAGTTCAGCGTGTCCGGCGAGGGCGAGGGCGATGCCACCTAC
GGCAAGCTGACCCTGAAGTTCATCTGCACCACCGGCAAGCTGCCCGTGCCCTGGCCCACCCTCGTGACCACCCTGACCTACGGCGTGCAG
TGCTTCAGCCGCTACCCCGACCACATGAAGCAGCACGACTTCTTCAAGTCCGCCATGCCCGAAGGCTACGTCCAGGAGCGCACCATCTTC
TTCAAGGACGACGGCAACTACAAGACCCGCGCCGAGGTGAAGTTCGAGGGCGACACCCTGGTGAACCGCATCGAGCTGAAGGGCATCGAC
TTCAAGGAGGACGGCAACATCCTGGGGCACAAGCTGGAGTACAACTACAACAGCCACAACGTCTATATCATGGCCGACAAGCAGAAGAAC
GGCATCAAGGTGAACTTCAAGATCCGCCACAACATCGAGGACGGCAGCGTGCAGCTCGCCGACCACTACCAGCAGAACACCCCCATCGGC
GACGGCCCCGTGCTGCTGCCCGACAACCACTACCTGAGCACCCAGTCCGCCCTGAGCAAAGACCCCAACGAGAAGCGCGATCACATGGTC
CTGCTGGAGTTCGTGACCGCCGCCGGGATCACTCTCGGCATGGACGAGCTGTACAAGGGAGGCTCCGGC*CTCGAG*AGCTACCCGATCTGG
TGGTCCTTGGCTGTGGGACCCCAGTACTCCTCTCTGAGCACTCAGCCCATTCTCTGTGCCAGCATCCCAGGCCTGGTACCGAAGCAGCTG
CGCTTCTGCAGGAACTACGTGGAGATCATGCCCAGCGTGGCTGAGGGTGTCAAAGCGGGCATCCAGGAGTGCCAGCACCAGTTCCGAGGC
CGGCGTTGGAACTGCACCACCGTCAGCAACAGCCTGGCCATCTTTGGCCCTGTTCTGGACAAAGCCACCCGGGAGTCAGCCTTTGTCCAT
GCCATCGCCTCCGCTGGAGTAGCTTTCGCAGTGACACGCTCCTGTGCAGAGGGATCAGCTGCTATCTGTGGGTGCAGCAGCCGCCTCCAG
GGCTCCCCAGGCGAGGGCTGGAAGTGGGGCGGCTGTAGTGAGGACATTGAATTTGGAGGAATGGTCTCTCGGGAGTTTGCCGATGCCAGG
GAGAACCGGCCGGATGCCCGCTCTGCCATGAACCGTCACAACAATGAGGCTGGGCGCCAGGCCATCGCCAGTCACATGCACCTCAAGTGC
AAATGCCACGGGCTATCTGGCAGCTGTGAAGTGAAGACCTGCTGGTGGTCGCAGCCGGACTTCCGCACCATCGGGGATTTCCTCAAGGAC
AAGTATGACAGTGCCTCGGAGATGGTGGTAGAGAAACACCGAGAGTCTCGTGGCTGGGTGGAGACCCTGAGGCCACGTTACACGTACTTC
AAGGTGCCGACAGAACGCGACCTGGTCTACTACGAGGCCTCACCCAACTTCTGCGAACCTAACCCCGAAACCGGCTCCTTCGGGACGCGT
GACCGCACCTGCAATGTGAGCTCGCATGGCATAGATGGGTGCGACCTGTTGTGCTGCGGGCGCGGGCATAACGCGCGCACTGAGCGACGG
AGGGAGAAATGCCACTGTGTTTTCCATTGGTGCTGCTACGTCAGCTGCCAGGAGTGCACACGTGTCTATGACGTGCACACCTGCAAGTAG

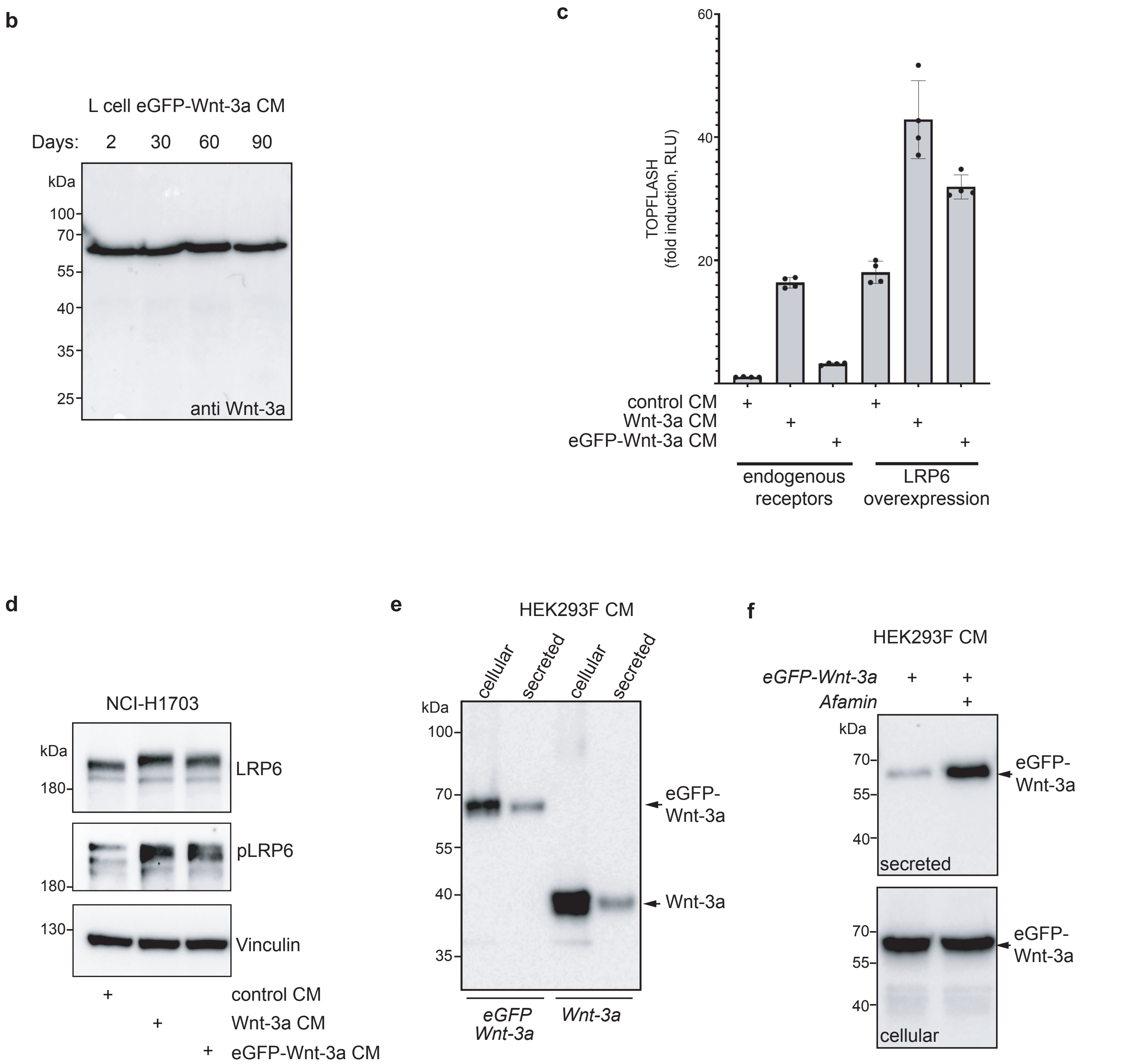

Supplementary Figure 1. Generation and functional analysis of eGFP-Wnt-3a. **a)** Sequence information for eGFP-Wnt-3a fusion protein. **b)** Western Blot analysis of eGFP-Wnt-3a present in CM from L cells. CM is stored at 4℃ after initial harvest and blue sepharose pull-down used to purify the Wnt-3a from CM for WB analysis at the indicated time periods. **c)** TOPFLASH TCF/LEF reporter assay showing the effect of LRP6 expression on the relative efficiency of eGFP-Wnt-3a to transduce Wnt/β-catenin signaling. Note that when cells overexpress LRP6, eGFP-Wnt-3a has an activity more similar to that of wild-type Wnt-3a. HEK293T cells in 96 wells were transfected with 20/2 ng TOPFLASH/Renilla, 20 ng *pCS2+ hLRP6*, 5 ng *MESD* and *LacZ* to bring total pDNA amount to 100 ng/well. 24 h post transfection cells were treated o/n with control CM, mWnt-3a CM or eGFP-mWnt-3a CM (form stable L-cells, 13 x conc). Error bars represent mean ± SD from 4 independent biological samples, represented as solid dots. The experiment was performed twice with similar results. **d)** LRP6 Western blot of endogenous LRP6 from lysates of NCI-H1703 cells treated with the indicated conditioned medium (CM) for 90 minutes. Note the characteristic upshift of the mature (upper) LRP6 protein band (top panel) and the corresponding increase in PPSP site phosphorylation (middle panel) upon addition of either Wnt-3a or eGFP-Wnt-3a CM. **e)** Example of Wnt-3a Western Blot analysis of cell lysates (cellular) or CM (secreted) from HEK293F suspension cells transfected with the indicated constructs. 20 µg of *Wnt-3a* or *eGFP-Wnt-3a* were transfected in 30 mL suspension cultures and incubated 4 days. **f)** Wnt-3a Western Blot analysis of cell lysates or CM (secreted) from HEK293F suspension cells transfected with the indicated constructs. 10 µg of *pCS2^{+}-eGFP-Wnt-3a* together with either 50 µg of *pCMV3* plasmid or *pCMV3-His-Afamin* were co-transfected in 60 mL HEK293F cultures.

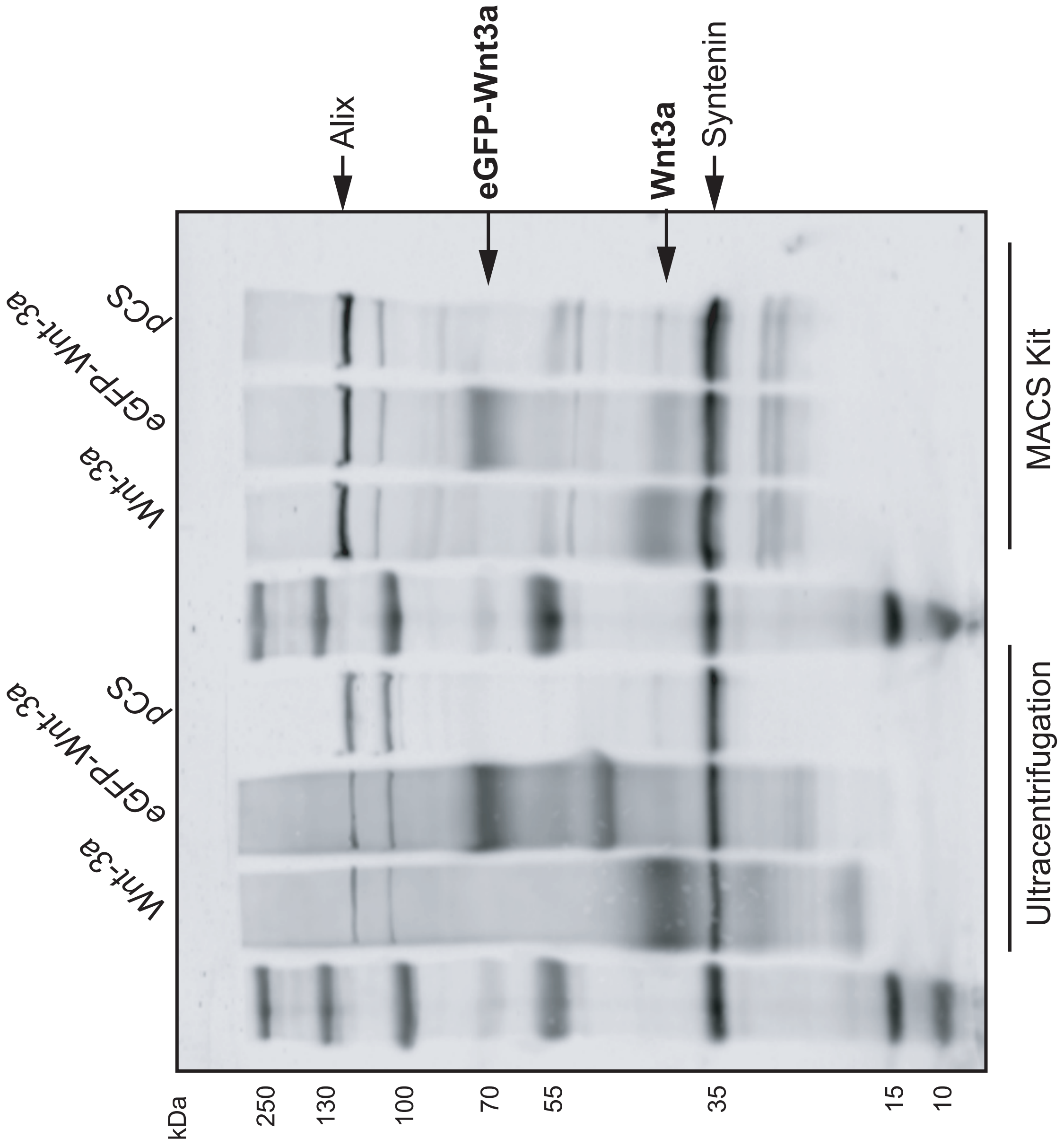
Alix
eGFP-Wnt3a
Wnt3a
Syntenin
pCS
eGFP-Wnt-3a
Wnt-3a
pCS
eGFP-Wnt-3a
Wnt-3a
MACS Kit
Ultracentrifugation
kDa
250
130
100
70
55
35
15
10

Supplementary Figure 2. Association of eGFP-Wnt-3a with exosomes. Western Blot analysis of exosomes purified from CM of HEK293F suspension cells transfected as indicated with Wnt-3a or eGFP-Wnt-3a. Exosomes were purified either by ultracentrifugation or MACS kit (see methods for details). The exosome marker proteins Alix and Syntenin as well as Wnt-3a and eGFP-Wnt-3a are indicated. M, molecular weight marker proteins.

a

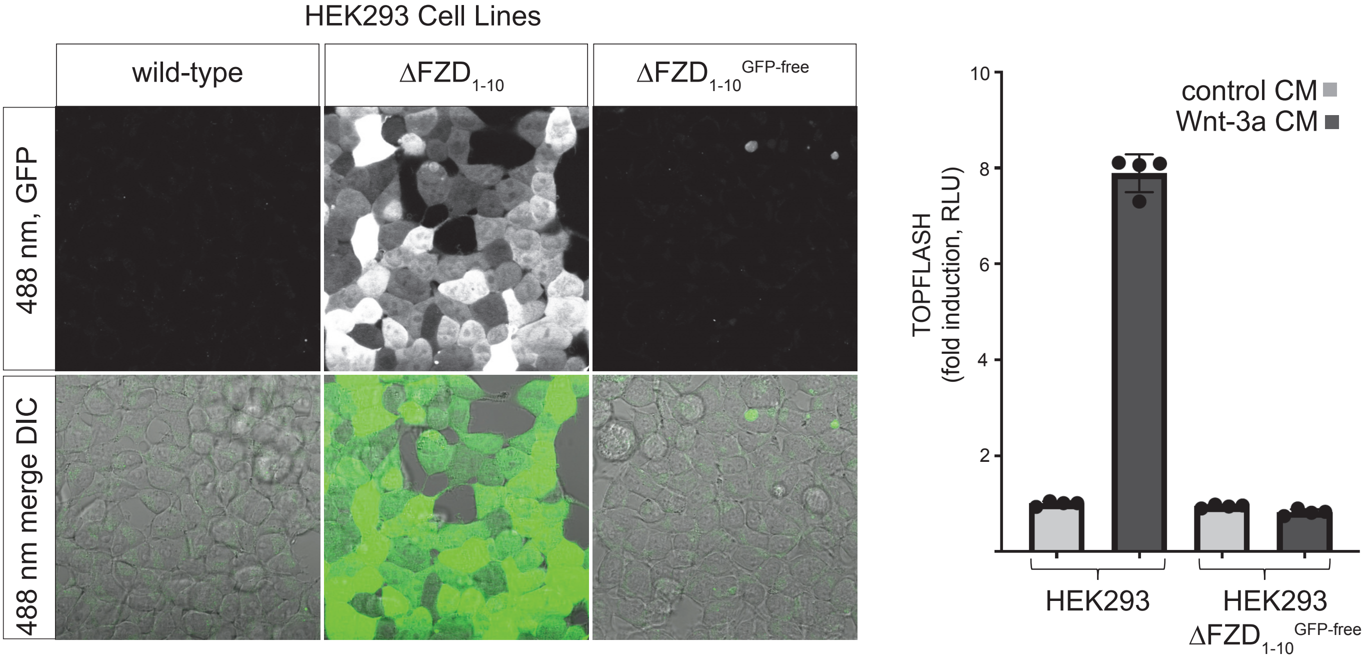

HEK293 Cell Lines
wild-type
ΔFZD1-10
ΔFZD1-10 GFP-free
488 nm, GFP
488 nm merge DIC
TOPFLASH (fold induction, RLU)
10
8
6
4
2
control CM
Wnt-3a CM
HEK293
HEK293 ΔFZD1-10 GFP-free

b

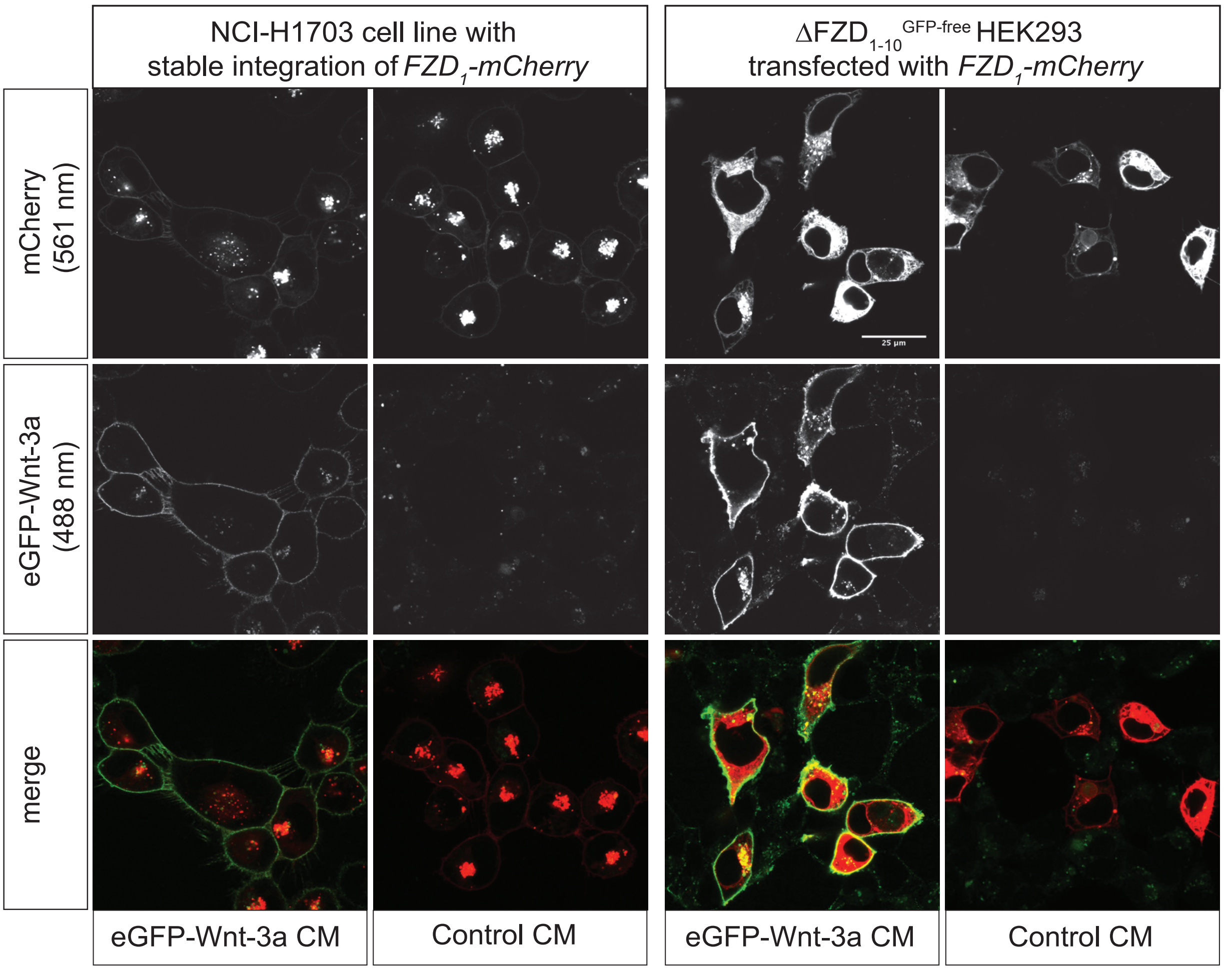

NCI-H1703 cell line with stable integration of FZD1-mCherry
ΔFZD1-10 GFP-free HEK293 transfected with FZD1-mCherry
mCherry (561 nm)
eGFP-Wnt-3a (488 nm)
merge
eGFP-Wnt-3a CM
Control CM
eGFP-Wnt-3a CM
Control CM

Supplementary Figure 3. CRISP/Cas9 mediated removal of GFP from ΔFZD$_{1\text{-}10}$ HEK293 cells and comparison of FZD$_1$-mCherry expression levels in transiently transfected ΔFZD$_{1\text{-}10}^{GFP\text{-}Free}$ and a stable FZD$_1$-mCherry expressing NCI-H1703 cell line. a) Fluorescence microscopy images of wild-type, ΔFZD$_{1\text{-}10}$ and ΔFZD$_{1\text{-}10}^{GFP\text{-}Free}$ HEK293 cells. The latter were generated by CRISPR/Cas9 mediated removal of the GFP gene present in the parental ΔFZD$_{1\text{-}10}$ cells[1]. The TOPFLASH TCF/LEF reporter assays shown on the right confirm the ΔFZD$_{1\text{-}10}^{GFP\text{-}Free}$ cells remain incapable of transducing Wnt-3a signals. Error bars represent mean ± SD from 4 independent biological samples represented as solid dots. The experiment was performed twice with similar results. **b)** Laser scanning confocal microscopy images showing a direct comparison of FZD$_1$-mCherry expression levels in ΔFZD$_{1\text{-}10}^{GFP\text{-}Free}$ HEK293 cells transiently transfected with *FZD$_1$-mCherry* and an NCI-H1703 cell line with stable integration of *FZD$_1$-mCherry* gene (top panels). The association of eGFP-Wnt-3a to these cells is also shown (middle panels). Note that, in order avoid strong overexposure of mCherry and GFP signals in the transiently transfected ΔFZD$_{1\text{-}10}^{GFP\text{-}Free}$ HEK293 cells, the laser intensity was reduced to levels where the FZD$_1$-mCherry expression on the NCI-H1703 stable cell line was barely detectable.

a Determination of eGFP-Wnt-3a concentration using GFP ELISA kit:

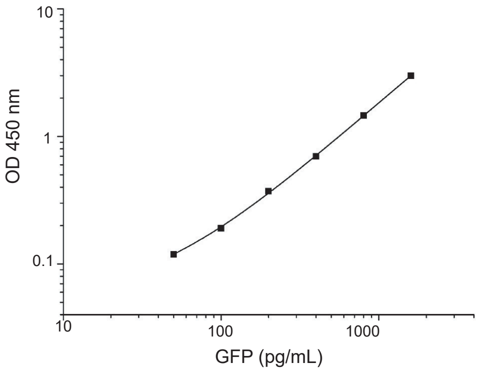

HEK293F suspension cell derived eGFP-Wnt-3a:

+ Afamin co-expression: 952 ± 68 ng/mL
- Afamin co-expression: 444 ± 63 ng/mL

L cell derived eGFP-Wnt-3a: 505 ± 24 ng/mL

Standard Curve Equation			
**Model**	Logistic		
**Equation**	Y=A2+(A1-A2)/(1+(X/X0)^p)		
**Reduced Chi-Sqr**	1.71143E-4		
**Adj.R-Square**	0.99986		
		Value	Std. Error
**B**	A1	0.05323	0.02156
**B**	A2	72.28422	169.32247
**B**	X0	27716.82	66533.037
**B**	p	1.10713	0.07886

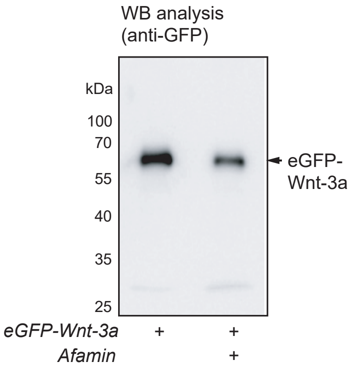

b Non-saturating binding of eGFP-Wnt-3a from L cell CM to Nluc-FZD$_4$:

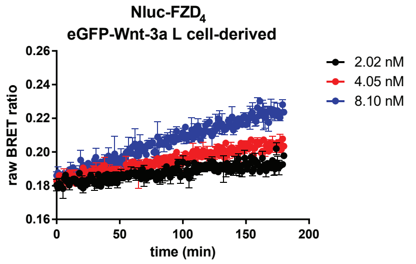

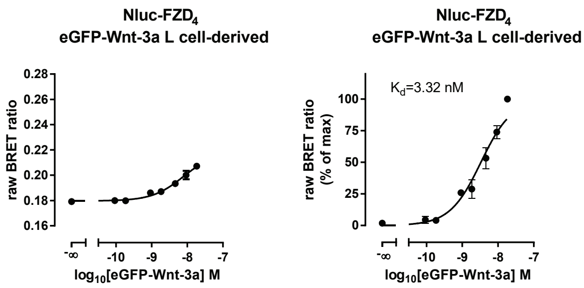

c Binding specificity of eGFP-Wnt-3a to Nluc-FZD$_4$:

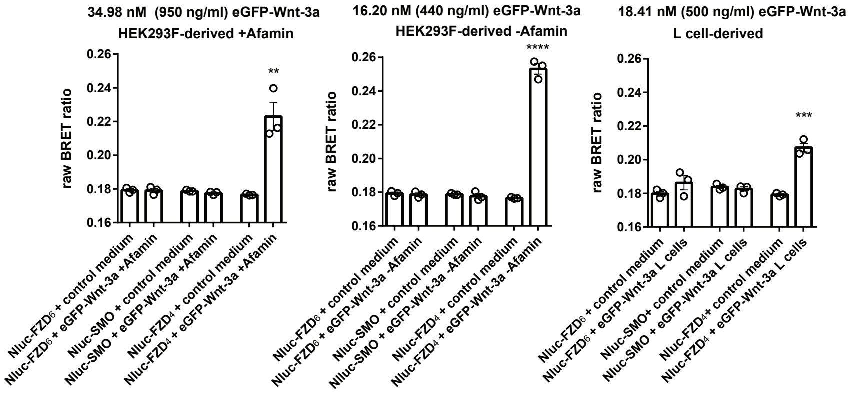

d Cell surface expression of the Nluc-tagged receptor constructs used in the study:

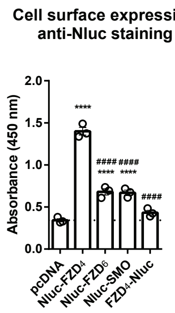

e

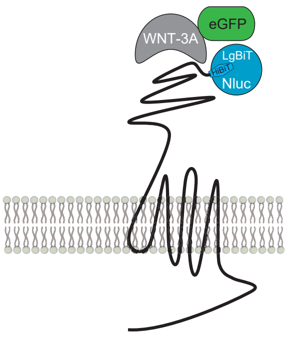

NanoBiT binding assay

f Nluc does not obstruct binding of eGFP-Wnt-3a to FZD$_6$

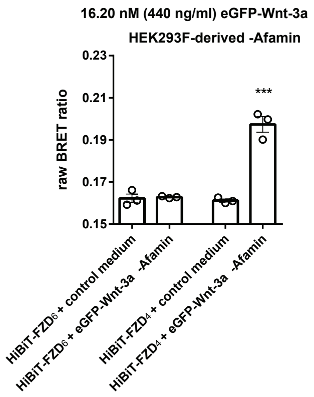

g Wnt-3a does not induce internalization of FZD$_4$

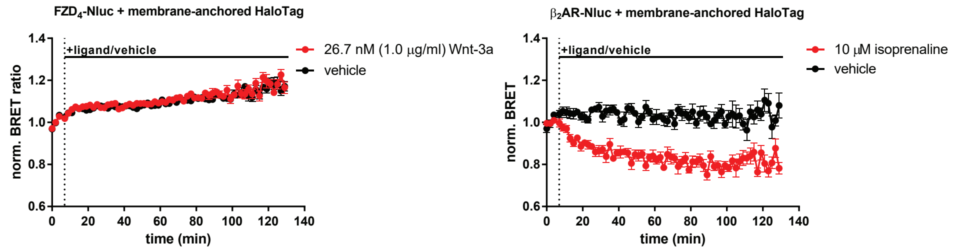

Supplementary Figure 4. Assessment of cell surface expression of the Nluc-tagged receptor constructs used in the study. Analysis of eGFP-Wnt-3a NanoBRET binding to Nluc-FZD_6 and Nluc-SMO. a) Details of GFP ELISA assay used to determine the concentration of eGFP-Wnt-3a present in the different CM preparations used for the experiment shown in Figure 5 as well as this Supplementary Figure. The panel on the right shows a WB analysis of eGFP-Wnt-3a from HEK293F suspension cells. **b)** L cell-derived eGFP-Wnt-3a binding to Nluc-FZD_4 does not reach saturation when a similar range of concentrations and the same incubation time were used as for the two HEK293F-derived preparations. The data in bar graphs represent mean ± S.E.M. of n=3 individual experiments. Kinetics are presented as mean ± S.D. from 3 independent experiments. **c)** Graphs present raw NanoBRET values obtained following 2 h incubation with each eGFP-Wnt-3a preparation (left – HEK293 cell-derived + Afamin; middle – HEK293-derived – Afamin; right – L cell-derived) in ΔFZD_{1-10} cells transiently overexpressing Nluc-FZD_6 or Nluc-SMO. (Nluc-FZD_4 data were taken from Fig. 5b and Supplementary Fig. 5c). No specific eGFP-Wnt-3a binding to either Nluc-FZD_6 or Nluc-SMO could be detected at the eGFP-Wnt-3a concentrations used. Data are presented as mean ± S.E.M. of n=3 individual experiments. **d)** Surface ELISA with anti-Nluc antibody was used to assess cell surface expression of Nluc-FZD_4, Nluc-FZD_6 and Nluc-SMO. The cells transfected with pcDNA and FZD_4-Nluc (C terminal, intracellular Nluc) served as the negative controls. The data represent mean ± S.E.M. of n=3 individual experiments; # represents comparison with Nluc-$FZD_{4,}$ * represents comparison with pcDNA. **e)** Schematic illustration of the NanoBiT BRET setup to detect eGFP-Wnt-3a binding to HiBiT-tagged receptors. **f)** eGFP-Wnt-3a does not bind to HiBiT-FZD_6 emphasizing that the Nluc tag is not obstructing the binding. HiBiT-tagging enables selective and simultaneous assessment of receptor cell surface expression and ligand-receptor interactions due to the cell impermeability of LgBiT complementary Nluc protein. The data represent mean ± S.E.M. of n=3 individual experiments **g)** Recombinant, human Wnt-3a does not induce receptor internalization that would compromise the assessments of ligand binding parameters in living cells. While β_2-adrenergic receptors (β_2AR-Nluc) expressed in ΔFZD_{1-10} HEK293 cells and stimulated with 10 µM isoprenaline readily internalized, FZD_4-Nluc did not internalize in response to 26.7 nM (1.0 µg/ml) of human Wnt-3a. The data represent mean ± SEM of n=4 individual experiments for FZD_4-Nluc and n=2 individual experiments for β_2AR-Nluc.
